# Framing social policy preferences: A scoping review and research agenda

**DOI:** 10.12688/openreseurope.21596.1

**Published:** 2025-12-01

**Authors:** Tijs Laenen

**Affiliations:** 1 Centre for Social Policy and Media, Movements & Politics, University of Antwerp, Faculty of Social Sciences, Antwerp, Flanders, 2000, Belgium

**Keywords:** social policy, public opinion, framing, priming, persuasion, survey experiment, political communication, political behavior

## Abstract

This article reviews over 160 studies that use survey experiments to analyze how framing affects citizens’ social policy preferences. In doing so, it evaluates three theoretical perspectives: one suggesting that such preferences are extremely malleable, another emphasizing their stability due to deeply rooted predispositions, and a third arguing that framing effects are contingent on multiple factors. Overall, the scoping review finds strong support for the contingency view, showing that framing effects depend on characteristics of the frames, receivers, senders, and context. However, because existing research has predominantly focused on the characteristics of frame receivers, significant gaps remain in our understanding of how framing effects are shaped by other contingency factors. Most importantly, few studies have examined how these factors *interact* in shaping framing effects on social policy preferences. Accordingly, a novel research agenda is proposed, emphasizing the need to study these contingency factors not in isolation, but in combination.

## Introduction

Citizens’ preferences are widely regarded as a cornerstone of social policymaking in welfare democracies ( Brooks & Manza, 2007). Understanding the extent to which these are influenced by elite framing of social policy is therefore essential. Against this backdrop, the present article takes stock of the extensive and expanding body of literature on how the framing of social policy in public, political, and media discourse influences citizens' policy preferences. By examining over 160 published studies that assess the causal impact of framing on social policy preferences through survey-embedded experiments, the aim is to evaluate three theoretical perspectives that have been prominently discussed in the literature. First, early scholars often assumed–either explicitly or implicitly–that citizens' social policy preferences are extremely malleable, swaying in whatever direction framing leads them. The underlying supposition here is that most citizens lack strong and stable opinions on many policy issues, which are often described as "non-attitudes" ( Converse, 1964). Others, by contrast, traditionally view these preferences as highly stable, arguing that people are predisposed by deeply rooted ideological beliefs and self-interest considerations, shaped through socialization. From this perspective, citizens are relatively immune to framing because their policy preferences are more or less fixed ( Sears & Funk, 1999). In between these extremes, a middle position has gradually developed, suggesting that while public opinion may indeed be susceptible to framing, its effects are strongly contingent on factors related to (1) the receiver of the frame (e.g., their partisanship), (2) the sender of the frame (e.g., source cues), (3) the frame itself (e.g., its strength), and (4) the context in which the frame is communicated (e.g., the information environment) ( Chong & Druckman, 2007; Zaller, 1992).

The main conclusion drawn from the scoping review reported in this article is that the extreme positions are misguided, as the influence of framing on social policy preferences is neither inherently strong nor inherently weak. Instead, the existing evidence clearly points more in the direction of the contingency view. However, several important knowledge gaps remain for future research. First, it is unclear how the characteristics of frame *senders* shape citizens’ susceptibility to the framing of social policy, as most prior research has focused exclusively on the characteristics of frame *receivers*, such as their political beliefs and sociodemographics. Second, while the attributes of the frames themselves have received significant scholarly attention, some important aspects–such as the format, frequency, and strength of different frames–remain under-studied in existing scholarship on the framing of social policy preferences. Third, relatively little is known about the influence of the broader context in which frames are delivered, including cross-country differences and over-time dynamics in framing effects. Finally, perhaps the most pressing need to advance existing scholarship is to investigate in much more detail how characteristics of the frame, sender, receiver, and context *interact* in shaping framing effects. Overall, these findings call for a nuanced understanding of the framing of social policy preferences, emphasizing that future research should account for the complex interplay between the factors that make such framing highly contingent.

In the following sections, I first discuss how framing is conceptualized in this article and delve deeper into the theories presented in existing literature to support the three perspectives on the influence of framing on (social) policy preferences. Next, I discuss the inclusion and exclusion criteria for the scoping review and provide a general overview of the studies included. Then, the results section is broken down into several parts, demonstrating that the impact of framing on social policy preferences is indeed influenced by characteristics of the frames, receivers, senders, and context. In doing so, I also identify the most significant blind spots in current research. Finally, I summarize the main findings and integrate them into a novel conceptual framework designed to guide future research on how framing affects popular preferences regarding social policy, which I argue is also relevant for research in other key and distinct policy domains, such as climate, defense, or immigration.

## Defining the concept of framing

Before examining the different theoretical views on the susceptibility of social policy preferences to framing, it is essential to first clarify how the concept of framing is understood within the context of this article. For the sake of comprehensiveness, I adopt a rather broad definition that includes different types of framing as recognized in framing theory, as well as the adjacent concepts of priming, persuasion, and information provision. There are two main reasons for adopting this inclusive approach. First, although these concepts clearly originate from distinct theoretical traditions, they are often used interchangeably in the literature on social policy preferences (e.g., Haeder *et al.*, 2023). Second, despite their theoretical differences, the concepts are often operationalized in strikingly similar ways in the studies reviewed here. For example, treatments that emphasize the ethnicity of policy recipients are described as “priming” in some studies (e.g., Ward & Denney, 2022), as “framing” in others (e.g., Doberstein & Smith, 2019) and as “information” in yet others (e.g., Peffley *et al.*, 1997), even though they rely on comparable experimental stimuli. Nevertheless, I briefly outline the different concepts here, highlighting how they are typically differentiated in the literature.

While many different definitions of framing exist, they typically refer to the way in which issues (in this case: social policies) are presented and emphasized in political communication, shaping how individuals perceive, interpret, and evaluate them. One key distinction in this literature is between equivalency and emphasis framing ( Scheufele & Iyengar, 2014). Equivalency framing, introduced by Tversky and Kahneman (1981), involves presenting logically identical content in different ways–such as emphasizing either gains or losses–which can lead to different judgments. Emphasis framing, on the other hand, involves selectively highlighting certain aspects of an issue while downplaying or omitting others, thereby influencing its perceived relevance or implications ( Chong & Druckman, 2007; Entman, 1993). Another important distinction is between episodic and thematic framing, as developed by Iyengar (1990). Episodic framing typically involves narrative accounts or personal stories that spotlight individual cases or events. Thematic framing, by contrast, presents issues in a more aggregated way, by showing broader trends and statistics. Priming, by comparison, typically refers to the process by which subtle exposure to certain cues influences the weight individuals assign to related considerations when forming judgments. While framing shapes how an issue is presented and interpreted, priming affects the salience of certain aspects of an issue, making them more prominent in individuals' minds. Persuasion, meanwhile, involves deliberate efforts to change attitudes or beliefs by presenting counter-arguments, usually with the aim of turning initial opponents of a policy into supporters (or *vice versa*). Finally, information provision refers to the communication of factual content intended to increase knowledge and thereby shift policy preferences. This can involve providing evidence-based information about social policies, thereby affecting how individuals understand and evaluate these policies.

Based on these considerations, I adopt a broad definition of framing as including *any emphasis placed on particular aspects of a social policy–including its design (e.g. financing), outcomes (e.g., poverty impact), recipients (e.g., their race) and context (e.g., demographic patterns)–that may influence citizens’ preferences regarding that policy.*

## Three views on the susceptibility of (social) policy preferences to framing

With this broad definition in mind, let us now turn to three theoretical perspectives that have figured most prominently in scholarly debates about the susceptibility of social policy preferences to framing. The first of these builds on the old idea that citizens generally do not possess strong and stable policy preferences. This notion can be traced back to the foundational work of Converse (1964), who argued that most citizens have ideologically inconsistent and highly unstable opinions on political issues, leading him to propose the existence of so-called “non-attitudes.” Although Converse was not very explicit about the influence of framing, he does imply that citizens often rely on cues and shortcuts provided by political elites to form policy preferences. If it is indeed true that citizens largely lack strong and stable preferences about social policy, then it seems likely that they will readily follow the framing of such policy in public, political, and media discourse.

The second theoretical perspective, by contrast, assumes that citizens’ political preferences are generally strong and stable. On the one hand, this argument posits that citizens tend to be predisposed by deeply rooted core ideological beliefs, shaped early on through primary socialization (within the family, school, and so on) and held consistently throughout their life course ( Sears & Funk, 1999). In this view, citizens’ attitudes towards social policy are tied to firmly held ideological beliefs ( Roosma & Laenen, 2023), thereby decreasing their short-term malleability in response to framing. On the other hand, citizens’ social policy preferences are not only predisposed through political socialization but also through experiences with, and expectations about, such policies. It is a classic thesis in welfare state literature that citizens’ preferences towards different social policies are shaped by their (perceived) self-interest in them, as both taxpayers and program beneficiaries ( Roosma & Laenen, 2023). From this perspective, it seems more likely that citizens first and foremost follow their vested self-interest rather than the framing of social policy in public, political, and media discourse. In sum, if most citizens are indeed ‘stuck in their beliefs’ due to ideological and self-interested predispositions, then their social policy preferences are unlikely to be influenced by framing.

A third and increasingly dominant perspective in the framing literature seeks to reconcile the two opposing extremes by emphasizing that the effects of framing are contingent upon a variety of factors. This more nuanced view has gained prominence since the seminal work of Zaller (1992), who underscored the importance of framing in shaping public opinion while also introducing a conditional model of how individuals form political preferences. Zaller’s Receive-Accept-Sample (RAS) model outlines three key steps through which frames influence attitudes: individuals must first receive the frame, then accept it, and finally sample it when forming or expressing opinions on political issues. Crucially, Zaller begins from the premise that citizens differ both in their motivation and in their ability to process political information. Even when individuals are exposed to a frame, Zaller argues that it will only influence their opinions if it is also accepted. Such acceptance is not automatic, but depends on people’s pre-existing beliefs. As Zaller (1992: 6) succinctly put it: “ *Every opinion is a marriage of information and predisposition: information to form a mental picture of the given issue, and predisposition to motivate some conclusion about it*.” This idea laid the groundwork for later research on directional motivated reasoning, which builds directly on Zaller’s model by proposing that individuals process information in a biased manner, favoring frames that confirm their prior (often partisan) beliefs ( Leeper & Slothuus, 2014; Taber & Lodge, 2006). More recently, scholars have also identified the possibility of so-called backfire or boomerang effects, whereby exposure to incongruent frames actually intensifies contrasting, pre-existing preferences ( Guess & Coppock, 2020; Nyhan & Reifler, 2010).

Beyond characteristics of those receiving the frames, additional research highlights the importance of other contingency factors. One line of work considers the characteristics of the frames themselves, including their content, direction, format, frequency and strength ( Chong & Druckman, 2007; Chong & Druckman, 2010; Entman, 1993; Scheufele & Iyengar, 2014; Soroka, 2014). This research starts from the assumption that not all frames are equally persuasive: some are simply more effective than others. In addition to frame features, studies have also explored the role of the characteristics of those sending the frames, such as the credibility of the source ( Druckman, 2001; Hartman & Weber, 2009). Finally, scholars are paying growing attention to the broader context in which frames are communicated. Although “context” is by nature a broad and somewhat diffuse concept, some authors have pointed to concrete factors such as the competitiveness of the information environment ( Chong & Druckman, 2013) or the salience of political issues ( Lecheler *et al.*, 2009), arguing that in real-world political communication, frames often compete with counter-frames and their impact can vary across contexts with different histories of public and political debates.

## Methodology

### Inclusion and exclusion criteria

To identify relevant studies for this scoping review, I employed a two-step sampling strategy. I first conducted a systematic search using academic databases–i.e., Google Scholar, Scopus, and Web of Science–based on keywords associated with the core concepts of framing (e.g., “framing”, “priming”, “information provision”, “persuasion”), social policy (e.g., “social policy”, “welfare state”, “unemployment benefits”, “healthcare”), and public opinion (e.g., “public opinion”, “public attitudes”, “policy preferences”, “mass attitudes”). This initial search was subsequently supplemented with snowball sampling, drawing on the bibliographies of retrieved studies to identify additional relevant contributions. From this pool, studies were screened based on a set of pre-defined eligibility criteria. Before discussing these, however, it is important to point out that the review process was ended after reaching theoretical saturation–that is, when continued inclusion of new studies no longer led to meaningful additions to the synthesis presented in this article. Saturation was assessed iteratively during the review process and was reached after reviewing well over 100 studies, at which point newly identified publications largely reiterated insights already captured. The list of studies discussed below is therefore not fully exhaustive, but captures the major conceptual and empirical contours of the field as it currently stands.

To be included, a study had to report findings from at least one survey-embedded *experiment* that allowed for the causal identification of framing effects on social *policy* preferences among members of the general *public*. This required, at a minimum, the application of at least one experimental treatment within a quantitative survey, and sufficient methodological transparency regarding the experimental design, meaning that the publication had to include detailed information about the treatments, outcomes, and procedures. Observational studies–such as those examining the impact of citizens’ media use–are thereby excluded from this review, as they typically lack the capacity to establish causal relationships in the way that experiments can. In terms of substantive focus, at least one of the outcome variables had to pertain to preferences concerning social policy, which is defined here as *the set of cash transfers and in-kind services aimed at achieving social objectives such as poverty alleviation, the reduction of income inequality, and the promotion of employment*. Accordingly, the review includes studies focusing on social policy in general as well as those examining the following specific policy domains: unemployment benefits, job training, healthcare, disability and sickness benefits, old-age pensions, elderly care, childcare, child benefits, means-tested social assistance (including U.S. “welfare”), universal basic income, social housing, housing benefits, education, and nutritional assistance (primarily “food stamps” in the U.S. context).

Furthermore, to ensure relevance to public opinion research, studies had to investigate the social policy preferences of ordinary citizens, rather than other relevant actors such as policy makers and administrators. Because the review is focused on the preferences of the general population, I also excluded all studies that are limited to student samples (e.g., Druckman *et al.*, 2012). Relatedly, a minimal sample size of 500 respondents was required for inclusion in the review. This threshold was chosen to mitigate excessive sampling error ( Kish, 1965) and to enable reliable subgroup analysis of treatment effect heterogeneity, which will emerge as a key theme in the literature that is not be ignored. Finally, additional criteria were set to ensure the quality and accessibility of the studies. Only peer-reviewed journal articles and book chapters published by academic presses were considered. Although this introduces the risk of publication bias, the peer review process was deemed essential to maintain scientific rigor. Notably, many of the included studies do report null effects, suggesting at least some tolerance for non-significant findings in this literature (see Table 1 for an overview).

**Table 1.** Overview of studies’ main framing effects and contingent framing effects.

	Main framing effects?	Framing contingent on frame receiver characteristics?	Framing contingent on frame sender characteristics?	Framing contingent on frame characteristics?	Framing contingent on frame context characteristics?
Frequencies (absolute & relative within column) yes (all effects are statistically significant) no (no significant effects) yes/no (some effects are significant, others not)	49 (31.4%) 14 (9.0%) 93 (59.6%)	44 (36.4%) 26 (21.5%) 51 (42.1%)	2 (40%) 1 (20%) 2 (40%)	11 (57.9%) 6 (31.6%) 2 (10.5%)	11 (34.4%) 7 (21.9%) 14 (43.7%)
Aarøe & Petersen, 2014	yes				no
Ashok & Huber, 2020	yes/no				
Asticher & Sager, 2025	no	no			no
Avdagic & Savage, 2021	yes	yes/no		yes	yes/no
Avdagic & Savage, 2024	no	yes/no		no	
Barabas *et al.*, 2020	yes	yes			
Barnes *et al.*, 2018	yes/no	yes/no			
Baumberg Geiger, 2021	yes/no	yes			yes/no
Baumberg Geiger, 2025	yes/no				
Baute *et al.*, 2022	yes	yes			yes/no
Bay & Pedersen, 2006	yes/no	yes/no			
Bay *et al.*, 2016	yes/no	yes			
Bendz & Oskarson, 2022	yes/no	yes			
Bendz & Oskarson, 2024	yes/no	no			
Berinsky, 2017	yes/no	yes/no		yes/no	yes
Bonoli *et al.*, 2024	yes/no				yes/no
Bor & Simonovits, 2021	no	no			
Bremer & Bürgisser, 2023	yes/no	yes/no			
Bridgman *et al.*, 2022	yes/no	no			
Brooks, 2012	yes/no				
Burgoon *et al.*, 2022	yes				yes/no
Buss, 2018	yes/no	yes			
Callaghan & Olson, 2017	yes	yes		no	
Campbell & Gaddis, 2017	yes/no	yes			
Cappelen & Midtbø, 2016	yes/no	no			
Carter, 2025	yes	yes/no			
Cattaneo *et al.*, 2020	yes	no			yes/no
Chueri *et al.*, 2025	yes/no	yes/no			yes/no
Cook *et al.*, 2010	yes/no				
Crabtree & Wehde, 2023	yes				
DeSante, 2013	yes/no	yes			
De Vries *et al.*, 2022	yes/no				
Doberstein & Smith, 2019	yes/no	no			
Epp & Jennings, 2020	yes/no	yes			
Ellis & Faricy, 2020	yes/no				
Ellis & Faricy, 2021	yes/no	yes			
Fang & Huber, 2019	yes/no	yes/no			
Fernández *et al.*, 2023	yes	yes/no			yes/no
Findor *et al.*, 2023	yes/no				
Finseraas *et al.*, 2017	yes/no				yes
Finseraas *et al.*, 2024	yes/no	yes/no			yes
Ford, 2015	yes/no	yes/no			
Fox *et al.*, 2022	yes?	no			
Gallego & Marx, 2017	yes/no	yes/no			
Gielens *et al.*, 2019	no?	yes/no			
Gift & Lastra-Anadon, 2023	yes	yes/no			
Gilens, 1996	no?				
Goerres *et al.*, 2020	yes/no	no			yes
Gollust & Lynch, 2011	yes/no	yes/no			
Gramacho *et al.*, 2025	yes/no				yes
Greenberg, 2018	yes/no	yes			no
Groskind, 1994; Groskind, 1991	yes	yes/no			
Gross & Wronski, 2021	yes/no				
Guardino & Mettler, 2020	Yes	yes			
Haderup Larsen & Schaeffer, 2021	yes/no				
Haeder, 2025a	yes/no	no			
Haeder, 2025b	yes/no	no			
Haeder *et al.*, 2023	yes/no	yes/no			
Haeder & Sylvester, 2024	yes/no	yes/no			
Hansen, 2007	yes/no	yes		yes	
Hansen, 2019	yes	yes/no			
Harell *et al.*, 2014	no	yes			
Harell & Lieberman, 2021	no	no			
Haselswerdt & Bartels, 2015	yes	yes			
Haselswerdt, 2020	yes	no			yes
Haselswerdt, 2022	yes/no	no			
Häusermann *et al.*, 2019	yes/no	yes/no			no
Hjorth, 2016	yes	yes			
Hopkins & Mummolo, 2017	yes/no			no	
Huber & Paris, 2013	yes/no				
Ingham & Levin, 2018	yes/no	yes			
Iyengar, 1990	yes				
Jacobsen, 2019	yes/no	yes/no		yes/no	
Jacobs & Matthews, 2012	yes	yes/no			
Jäger, 2024	no	yes			yes
Jensen & Kevins, 2019	yes/no	yes			
Jensen & Petersen, 2017	yes	yes/no			no
Jerit, 2009	yes/no	yes		yes	
Johnson *et al.*, 2023	yes/no	yes/no			
Jordan *et al.*, 2022	yes/no	yes		yes	
Kawata *et al.*, 2024	yes	yes/no		yes	
Kangas, 1997	yes/no				
Kangas *et al.*, 2022	yes/no	yes			
Kangas *et al.*, 2014	yes	yes/no			
Karra & Sandoe, 2020	yes				
Keiser & Miller, 2019	yes	yes			
Kootstra, 2016	yes				
Kootstra, 2017	yes				
Kootstra & Roosma, 2018	yes	yes/no			yes/no
Kuklinski *et al.*, 2000	no	no			
Kuhn & Kamm, 2019	yes	yes/no			yes
Kumlin, 2011	yes/no	yes/no	yes	yes	
Kumlin & Goerres, 2022	yes/no	yes	yes		
Kumlin & Nemčok, 2024	yes/no	yes/no			
Kuziemko *et al.*, 2015	yes/no				no
Kweon & Choi, 2022	yes	yes/no			
Laenen, 2023	yes/no	yes/no		yes	yes/no
Laenen *et al.*, 2023	yes/no	yes			
Laenen, 2025	yes/no				yes/no
Laenen & Aerts, forthcoming	yes/no	yes			
Lawrence & Sides, 2014	no				
Lergetporer *et al.*, 2018	yes	yes/no			no
Lergetporer *et al.*, 2020	yes/no	yes/no			
Levy, 2021	yes/no				
Lindner *et al.*, 2023	no	yes/no			
Linden & Reibling, 2022	yes/no	no			
Liscow & Pershing, 2022	yes/no				
Lim & Tanaka, 2019	yes	yes			
López-Santana *et al.*, 2023	no				
Magni, 2024	yes/no	no			
Martinangeli & Windsteiger, 2023	yes/no	yes/no			
Marx & Schumacher, 2016	yes	yes			
McIntyre *et al.*, 2024	yes/no	yes/no		no	yes
Mulayi *et al.*, 2023	yes	yes/no			
Muñoz & Pardos-Prado, 2019	yes/no	yes			
Myers *et al.*, 2024	yes/no	yes			
Naumann, 2017	yes/no	yes			
Naumann, 2018	yes/no	yes			
Naumann *et al.*, 2020	yes				yes/no
Naumann *et al.*, 2024	yes	yes			
Nicholson-Crotty *et al.*, 2021	yes/no	yes			
Palmer, 2018				yes	
Pedersen & Larsen, 2019	yes	yes/no		yes	
Pedersen, 2019	yes	yes/no		yes	
Peffley *et al.*, 1997	yes/no	yes			
Perna & de Reguero, 2025	yes				yes
Petersen *et al.*, 2010	yes/no	no			
Ponza *et al.*, 1988	yes	yes/no			
Porumbescu *et al.*, 2025	yes	no		yes	
Qi & Haselswerdt, 2024	no	yes/no			
Reeskens & van der Meer, 2017; Reeskens & van der Meer, 2018	yes				
Reeskens *et al.*, 2021	yes	yes/no			
Rincón, 2021	yes/no				
Rincón *et al.*, 2022	yes/no				
Rincón, 2024		yes/no			
Rodriguez *et al.*, 2010	no	no			
Sahn *et al.*, 2025	no	no		no	
Sawler, 2025	yes/no				
Schaeffer & Haderup Larsen, 2023	yes	yes/no			
Schueler & West, 2016	yes	yes			
Schuetz *et al.*, 2023	yes	yes			yes
Senghaas *et al.*, 2022a	yes				
Senghaas *et al.*, 2022b	yes/no				
Sievert & Bruder, 2024	yes/no				
Slothuus & de Vreese, 2010	yes/no	yes	yes/no		
Smith, 1987	yes	no			
Song *et al.*, 2024		yes			
Stadelmann-Steffen & Dermont, 2020	yes/no	no			yes/no
Stanley & Hartman, 2018	yes/no				
Stenderup & Pedersen, 2025	yes/no	yes/no			
Tesler, 2012	no	yes/no	yes/no		
Thomas *et al.*, 2023		yes			
Thorp & Larner, 2025	yes/no	yes/no			
Thorson & Trump, 2024	yes/no	yes/no			
Toossi, 2022	yes	yes			
van den Heijkant *et al.*, 2024	yes	yes/no	no		
van der Meer & Reeskens, 2021					yes/no
Wang, 2023	yes/no			no	
Ward & Denney, 2022	yes/no	no			
Wetts & Willer, 2018	yes/no	no			
Will, 1993	yes/no				
Wu, 2021	yes/no				
Yang & Hopkins, 2025	yes/no	yes			
Yeung, 2022		no			
Zhirkov *et al.*, 2025	yes/no	Yes			

### Overview of studies included

The scoping review includes a total of 167 studies, with some reporting a single experiment and others multiple experiments within one publication. Geographically, there is a clear bias towards Europe and the US, with almost no studies conducted outside these regions. Notable exceptions include Japan and South Korea, with 3 and 4 studies, respectively (e.g., Kweon & Choi, 2022; Ward & Denney, 2022). Within Europe, Western European countries clearly dominate, with Scandinavia (i.e., Denmark, Finland, Norway, and Sweden), continental Europe (i.e., Belgium, the Netherlands, and especially Germany) and the United Kingdom comprising almost the total number of studies included. Southern and Eastern European countries are less frequently represented, and when included, they are usually part of larger cross-national experiments (Spain being the exception). With regard to the time period, most of the experiments included in this review were conducted relatively recently, with the majority dating from after 2010.

The majority of the experiments included in this review were conducted on non-probability-based samples of the general population, typically sourced from (mostly online) opt-in panels such as Amazon’s Mechanical Turk (e.g., Wu, 2021), Bilendi (e.g., Laenen, 2025) and YouGov (e.g., Baumberg Geiger, 2021). A relatively smaller number of studies report the results of survey experiments conducted among samples recruited via probability-based (online) panels, such as the German Internet Panel (GIP) and the Longitudinal Internet Studies for the Social Sciences (LISS) panel in the Netherlands (e.g., Buss, 2018; Lindner *et al.*, 2023, respectively). Both types of samples are included in this review, as recent methodological research suggests that the observed experimental treatment effects are usually highly comparable ( Coppock *et al.*, 2018; Mullinix *et al.*, 2015). With regard to sample size, there is considerable variation across the studies, although most experiments included between 1000 and 4000 respondents.

Most reported experiments are classic between-subjects, split-sample designs that divide the full sample into a number of treatment groups and possibly a control group, with the number ranging between a minimum of 2 (e.g., Bendz & Oskarsson, 2022) and a maximum of 9 (e.g., Gift & Lastra-Anadon, 2023) split samples. This is followed by factorial conjoint and vignette experiments, which administer multiple experimental treatments simultaneously, primarily focusing on the impact of framing individual social policy recipients or policy design features. There are only a few genuine within-subject experiments included in the review, all of which assess how framing affects individual opinion change regarding social policy ( Guardino & Mettler, 2020; Johnson *et al.*, 2023; Laenen & Aerts, forthcoming; Schuetz *et al.*, 2023).

In terms of dependent variables, most studies focus on measurements of stated policy support, which includes support for existing social policy programs (e.g., the Earned Income Tax Credit in the US; Callaghan & Olson, 2017), reforms thereof (e.g., increasing the age required to qualify for pensions; Ingham & Levin, 2018), or entirely new policies (e.g., universal basic income; Laenen, 2023). Other studies examine preferences for increasing, decreasing, or stabilizing spending on social policy (e.g., Lergetporer *et al.*, 2018) or preferred benefit levels (e.g., Linden & Reibling, 2022). Additionally, some studies explore support for the imposition of social obligations, such as work-related requirements (e.g., Gielens *et al.*, 2019), benefit sanctions (e.g., Kootstra & Roosma, 2018), and administrative burdens (e.g., Haeder & Sylvester, 2024). Beyond direct policy support, some studies investigate dependent variables closely related to it, such as the level of satisfaction or trust in social policy (e.g., Bendz & Oskarsson, 2024), its perceived sustainability (e.g., Goerres *et al.*, 2020), or the perceived deservingness of policy recipients (e.g., Crabtree & Wehde, 2023).

In terms of independent variables, all studies included in this review focus on the effects of some form of framing on social policy preferences. However, there is variation in the framing concepts used, and the categories of framing content under consideration. While a large number of studies do not specify the exact framing concept (e.g., Aarøe & Petersen, 2014), those that do refer explicitly to framing itself (e.g. Avdagic & Savage, 2021) and/or adjacent constructs such as information provision (e.g., Qi & Haselswerdt, 2024), priming (e.g., Haselswerdt, 2020), and persuasion (e.g., Kawata *et al.*, 2024). Regarding the framing content, I distinguish four broader categories considered in the literature, referring to: (1) policy recipients, (2) policy design, (3) policy outcomes, and (4) policy context. The framing of policy recipients–either episodically as individual claimants (e.g., Bonoli *et al.*, 2024) or thematically as a group (e.g., Kootstra, 2017)–is most common in the literature, followed by the framing of policy design (e.g., Sievert & Bruder, 2024), policy outcomes (e.g., Laenen & Aerts, forthcoming) and policy context (e.g., Naumann, 2017). I will return to the specifics of the different categories below.

## Results

A quick glance at Table 1 already shows that framing effects on social policy preferences are neither inherently strong nor inherently weak. Instead, the available evidence lends strong support to the contingency view, which has become dominant in the broader framing literature.

There are two main reasons for this. First, the second column of the table, which reports the results regarding the main effects of the experimental treatments of each study, clearly illustrates that framing effects vary considerably across studies. About 30% of the studies unequivocally find statistically significant effects of framing on social policy preferences (indicated as “yes” in Table 1). In some cases, these effects are quite large. For instance, when 70% of respondents change their initial opinion on a universal basic income after learning about its projected impact on their household income and poverty levels ( Laenen & Aerts, forthcoming). In other instances, framing effects are statistically significant but modest in size. For example, when opposition to raising the retirement age declines from 38.3% to 33.4% after exposure to a frame emphasizing the need for pension reform ( Naumann, 2017). Conversely, some studies find no significant main effects of framing at all (9%, indicated as “no” in Table 1). For example, when attitudes reflecting welfare chauvinism generally remain unchanged in response to immigration framing ( Avdagic & Savage, 2024). Most commonly, however, findings are mixed, with some frames producing significant main effects, while others in the same study do not (about 60%, indicated as “yes/no” in Table 1). For instance, when framing the health status of benefit recipients affects support for disability benefits, whereas framing their race does not ( Fang & Huber, 2019).

Second, the next four columns in Table 1 show that framing effects are indeed often (but not always) contingent on characteristics of the frame itself, the receiver, the sender, and the broader context in which it was communicated. The table also highlights that most previous studies have concentrated on how framing effects vary by receiver characteristics, primarily by analyzing treatment heterogeneity across ideological and sociodemographic subgroups within the survey sample. Here too, the overall picture is mixed: some studies (36.5%) report heterogeneous effects, others find uniform effects (21.5%), and many identify both (42%). Despite the clear emphasis on frame receivers, it is important to note that Table 1 highlights only the relatively limited number of studies that explicitly and systematically examine how features of the frame, sender, or context–such as frame direction, source credibility, or the information environment–influence the framing of social policy preferences. However, this does not mean that these factors had no influence on the results in experiments where they were not directly manipulated. On the contrary, they may have shaped outcomes in indirect or unobserved ways.

In what follows, I review the existing research on the influence of each contingency factor individually and, along the way, consider the relatively limited number of studies that explore their potential interactions in shaping citizens’ social policy preferences.

### The influence of frame characteristics

In this section, I examine the existing body of evidence concerning the influence of the characteristics of frames themselves. More specifically, I focus on five key characteristics: frame content, direction, format, strength, and frequency–all of which have been recognized as important in the broader framing literature, as noted above.

I begin by examining the impact of frame content, which is addressed–often implicitly–in nearly every study included in this review. To structure the discussion, I draw on the four content categories introduced earlier: policy recipients, policy design, policy outcomes, and policy context. Before exploring these categories in detail, I briefly define each and consider the relative influence they exert on social policy preferences, as reported in existing research. The first category, *policy recipients*, includes studies that examine how citizens’ preferences are shaped by the framing of different features of the (potential) beneficiaries of social policies, such as their age, ethnicity, or work history. The second category, *policy design*, encompasses studies that investigate the influence of highlighting certain design features–such as the generosity and conditionality of social policies–on citizens’ attitudes toward such policies. Third, *policy outcomes* refers to research assessing how framing the (anticipated) effects of policies on outputs like poverty or employment influences preferences. Finally, *policy context* includes studies that explore how the framing of the broader economic, demographic, or socio-political environment in which policies are implemented affects public attitudes.

Table 2 presents the distribution of the reviewed studies across these categories and indicates whether they report significant main treatment effects. The findings do not reveal clear overall patterns regarding the relative impact of the different categories. Instead, the evidence across columns is mixed, yielding a nuanced and at times contradictory picture. This impression is supported by the few studies that directly compare the effects of different content categories, which point in divergent directions. For example, while some studies suggest that the framing of policy design features has a greater impact than that of policy recipients ( Laenen, 2025; Palmer, 2018), others draw the opposite conclusion ( Ellis & Faricy, 2020; Muñoz & Pardos-Prado, 2019), or find both to be equally influential ( Wu, 2021). Similar patterns emerge for other category comparisons, leaving open the question of which type of framing content is most effective for further investigation.

**Table 2.** Overview of frame content categories.

	Policy recipients - individual	Policy recipients - group	Policy design	Policy outcomes	Policy context
Frequencies (absolute & relative within column) yes (all effects are statistically significant) no (no significant effects) yes/no (some effects are significant, others not)	19 (34.5%) 3 (5.5%) 33 (60.0%)	11 (61.1%) 1 (5.6%) 6 (33.3%)	14 (32.5%) 3 (7.0%) 26 (60.5%)	15 (45.5%) 4 (12.1%) 14 (42.4%)	12 (32.4%) 10 (27.0%) 15 (40.6%)
Aarøe & Petersen, 2014	yes				
Ashok & Huber, 2020			yes	yes/no	
Asticher & Sager, 2025				no	
Avdagic & Savage, 2021					yes
Avdagic & Savage, 2024					no
Baumberg Geiger, 2021	yes/no				
Baumberg Geiger, 2025	yes/no				
Baute *et al*., 2022			yes		
Bay & Pedersen, 2006			yes/no		
Bay *et al*., 2016			yes/no		
Bendz & Oskarson, 2022					yes/no
Bendz & Oskarson, 2024			yes/no	yes/no	
Bonoli *et al*., 2024	yes/no				
Bor & Simonovits, 2021	no				
Bremer & Bürgisser, 2023			yes/no		
Bridgman *et al*., 2022	yes/no				
Brooks, 2012		yes/no			no
Burgoon *et al*., 2022			yes		
Buss, 2018	yes/no				
Callaghan & Olson, 2017		Yes	yes		
Campbell & Gaddis, 2017			yes/no		
Cappelen & Midtbø, 2016			yes/no		
Carter, 2025			yes	yes	
Chueri *et al*., 2025	yes/no				
Cook *et al*., 2010			yes/no		
Crabtree & Wehde, 2023		Yes			
DeSante, 2013	yes/no				
De Vries *et al*., 2022	yes/no				
Doberstein & Smith, 2019	yes/no				
Epp & Jennings, 2020		Yes		yes/no	
Ellis & Faricy, 2020		yes/no	yes/no		
Ellis & Faricy, 2021			yes/no		
Fang & Huber, 2019	yes/no			yes	
Fernández *et al*., 2023			yes		
Findor *et al*., 2023			yes/no		
Finseraas *et al*., 2017			yes/no		
Finseraas *et al*., 2024					yes/no
Ford, 2015	yes/no				
Fox *et al*., 2022	yes				
Gallego & Marx, 2017			yes/no		
Gift & Lastra-Anadon, 2023	yes				
Gilens, 1996	yes/no				
Goerres *et al*., 2020					yes/no
Gollust & Lynch, 2011	yes/no				
Greenberg, 2018	no				
Groskind, 1994; Groskind, 1991	yes		yes	yes/no	
Gross & Wronski, 2021	yes/no				
Guardino & Mettler, 2020			yes	yes	
Haderup Larsen & Schaeffer, 2021	yes/no				
Haeder, 2025a	yes/no				
Haeder, 2025b	yes/no				
Haeder *et al*., 2023	yes/no				
Haeder & Sylvester, 2024			yes/no		
Hansen, 2019	yes	Yes			
Harell *et al*., 2014	no				
Harell & Lieberman, 2021					no
Haselswerdt & Bartels, 2015			yes	yes	
Haselswerdt, 2020					yes/no
Haselswerdt, 2022			yes/no		
Hausermann *et al*., 2019			yes/no		
Hjorth, 2016	yes				
Hopkins & Mummolo, 2017					yes/no
Iyengar, 1990	yes				
Jacobsen, 2019				yes/no	
Jacobs & Matthews, 2012				yes	
Jäger, 2024					no
Jensen & Petersen, 2017	yes	Yes			
Jerit, 2009				yes/no	
Johnson *et al*., 2023				yes/no	
Jordan *et al*., 2022				yes/no	
Kawata *et al*., 2024	yes	Yes			
Kangas *et al*., 2014				yes	
Keiser & Miller, 2019			yes		
Kootstra, 2016	yes				
Kootstra, 2017		Yes			
Kootstra & Roosma, 2018				yes	
Kuklinski *et al*., 2000		No	no		
Kuhn & Kamm, 2019		Yes			
Kumlin, 2011				yes	
Kumlin & Goerres, 2022					yes/no
Kumlin & Nemčok, 2024					yes/no
Kuziemko *et al*., 2015	yes/no			yes	no
Kweon & Choi, 2022		Yes			
Laenen, 2023			yes/no	yes/no	
Laenen *et al*., 2023			yes/no		
Laenen, 2025		yes/no	yes/no		
Laenen & Aerts, forthcoming				yes/no	
Lawrence & Sides, 2014					no
Lergetporer *et al*., 2020				yes/no	yes/no
Levy, 2021	yes/no				
Lindner *et al*., 2023					no
Linden & Reibling, 2022	yes/no				
Liscow & Pershing, 2022				yes/no	
Lim & Tanaka, 2019			yes	yes	
López-Santana *et al*., 2023			no		
Magni, 2024	yes/no				
Marx & Schumacher, 2016					yes
Martinangeli & Windsteiger, 2023					yes/no
Muñoz & Pardos-Prado, 2019		Yes	yes/no		
Myers *et al*., 2024	yes/no				
Naumann, 2017					yes/no
Naumann *et al*., 2020	yes				
Naumann *et al*., 2024	yes				
Nicholson-Crotty *et al*., 2021			yes/no		
Palmer, 2018		yes/no		yes/no	
Pedersen & Larsen, 2019				yes	
Pedersen, 2019			yes		
Peffley *et al*., 1997		yes/no			
Perna & de Reguero, 2025	yes				
Petersen *et al*., 2010	yes/no				
Ponza *et al*., 1988	yes				
Porumbescu *et al*., 2025			yes		
Qi & Haselswerdt, 2024			no	no	
Reeskens & van der Meer, 2017; Reeskens & van der Meer, 2018	yes				
Reeskens *et al*., 2021	yes				
Rincón, 2021			yes/no		
Rincón *et al*., 2022			yes/no		
Rodriguez *et al*., 2010				no	no
Sahn *et al*., 2025				no	no
Sawler, 2025	yes/no				
Schaeffer & Haderup Larsen, 2023	yes				
Schuetz *et al*., 2023					yes
Senghaas *et al*., 2022a	yes				
Senghaas *et al*., 2022b	yes/no				
Sievert & Bruder, 2024			yes/no		
Stadelmann-Steffen & Dermont, 2020			yes/no		
Stenderup & Pedersen, 2025			yes	yes	
Thorp & Larner, 2025	yes/no				
Thorson & Trump, 2024					yes/no
Toossi, 2022		Yes			
Wang, 2023	yes/no				
Ward & Denney, 2022	yes/no				
Wetts & Willer, 2018		yes/no			yes
Will, 1993	yes/no				
Wu, 2021	yes/no		yes/no		
Yang & Hopkins, 2025			yes/no		
Zhirkov *et al*., 2025	yes/no				

*Note: the table only discusses main treatment effects.

Let us first delve deeper into the content category of policy recipients, which has garnered the most consideration in the literature. As shown in Table 2, I distinguish between more episodic framings of recipients at the individual level and more thematic framings at the group level. I will return to this distinction later when discussing the level of framing. For now, I focus on the different types of policy recipient features considered in the experimental studies included in this review (see Table 3 for an overview). Arguably, the most attention has been devoted to the race or ethnicity of recipients–reflecting, in part, the U.S.-centric nature of the literature. A longstanding hypothesis, informed by several theoretical perspectives–including welfare deservingness theory ( van Oorschot, 2000) and social identity theory ( Tajfel & Turner, 1979)–holds that (majority-group) citizens express more negative attitudes toward social policies when recipients are described as belonging to ethnic or racial minorities. However, Table 3 suggests that empirical support for this claim is more limited than often assumed. In fact, the evidence appears roughly balanced: while 23.5% of the studies supports the hypothesis (e.g., Callaghan & Olson, 2017), 36.5% clearly refutes it (e.g., Peffley *et al.*, 1997), and 40% presents a more nuanced picture–for instance, showing that some racial cues are effective while others are not (e.g., distinguishing between Blacks and Hispanics in the U.S. context; Wang, 2023), that effects depend on the specific outcome measure (e.g., perceived deservingness versus policy support; Gilens, 1996), or that race only matters in interaction with other recipient characteristics (e.g., their work history; DeSante, 2013). Moreover, these effects tend to be relatively modest compared to those associated with other recipient characteristics. For instance, when German citizens are asked to indicate their preferred level of old-age pensions, the impact of claimants’ ethnicity is notably smaller than the impact of their income and contribution record ( Naumann *et al.*, 2024). In addition, the effects of ethnic or racial cues are often concentrated within specific subgroups of the population–such as individuals high in racial resentment (e.g., Zhirkov *et al.*, 2025)–, as I will discuss in more detail below. By contrast, there is considerably stronger evidence that preferences become more negative when recipients are framed as (newly arrived) immigrants, regardless of their race (e.g., Magni, 2024). Exceptions to this pattern are relatively rare and occur mainly in the U.S. context (e.g., Levy, 2021), suggesting that citizens may respond more strongly to perceived violations of the principle of reciprocity than to a lack of shared identity per se ( Kootstra, 2016; Kootstra, 2017).

**Table 3.** Overview of policy recipient features.

	Race/ethnicity	Immigration status	Religion	Gender	Age	Work/contribution record	Employment status	Education	Social class	Financial situation	Household composition	Health	Cause of social risk	Motivation/efforts
Frequencies (absolute and relative within columns) Yes no yes/no	7 (23.3%) 11 (36.7%) 12 (40.0%)	11 (57.9%) 2 (10.5%) 6 (31.6%)	3 (75%) 0 (0%) 1 (25%)	8 (50.0%) 5 (31.2%) 3 (18.8%)	6 (54.5%) 1 (9.1%) 4 (36.4%)	9 (81.8%) 0 (0%) 2 (18.2%)	11 (100%) 0 (0%) 0 (0%)	2 (75%) 0 (0%) 1 (25%)	2 (40%) 1 (20%) 2 (40%)	9 (90%) 1 (10%) 0 (0%)	10 (58.2%) 0 (0%) 7 (41.2%)	9 (75.0%) 2 (16.7%) 1 (8.3%)	11 (91.7%) 0 (0%) 1 (8.3%)	15 (88.2%) 1 (5.9%) 1 (5.9%)
Aarøe & Petersen, 2014						yes								yes
Baumberg Geiger, 2021	yes			no	yes/no	yes/no						yes	yes	
Bonoli *et al*., 2024		yes/no		no		yes	yes			yes	yes			no
Bridgman *et al*., 2022	no	yes/no		no			yes			yes	yes/no	yes		
Brooks, 2012	yes/no	yes/no							yes/no					
Buss, 2018	yes/no				yes/no						yes/no		yes	yes
Callaghan & Olson, 2017	yes													
DeSante, 2013	yes/no													yes
De Vries *et al*., 2022	no			no	yes				yes/no					
Doberstein & Smith, 2019	no			yes										
Ellis & Faricy, 2020							yes		yes					
Fang & Huber, 2019	no											yes		
Ford, 2015	yes/no	yes	yes											
Fox *et al*., 2022														
Gielens *et al*., 2019		no												
Gift & Lastra-Anadon, 2023						yes						yes		yes
Gilens, 1996	yes/no													
Gollust & Lynch, 2011	no								yes				yes	
Greenberg, 2018	no													
Gross & Wronski, 2021	no												yes	
Haderup Larsen & Schaeffer, 2021		yes	yes		yes				no					
Haeder, 2025a	no						yes				yes	yes		
Haeder, 2025b	no						yes				yes	yes		
Haeder *et al*., 2023		yes/no			no					no		no		
Hansen, 2019													yes	yes
Harell *et al*., 2014	no													
Hjorth, 2016		yes									yes			
Jensen & Petersen, 2017							yes					yes		yes
Kootstra, 2016	yes/no	yes		yes		yes					yes			yes
Kootstra, 2017	yes	yes	yes											
Kweon & Choi, 2022										yes				yes
Levy, 2021		yes/no												
Linden & Reibling, 2022	yes				yes						yes/no		yes/no	yes
Magni, 2024		yes	yes/no			yes		yes/no			yes	yes		yes
Muñoz & Pardos-Prado, 2019		yes												
Myers *et al*., 2024	yes/no	yes/no		yes/no			yes				yes/no			yes
Naumann *et al*., 2020					yes									
Naumann *et al*., 2024	yes			yes/no		yes				yes	yes			
Palmer, 2018	yes/no													
Peffley *et al*., 1997	no							yes						yes
Perna & de Reguero, 2025		yes					yes						yes	
Reeskens & van der Meer, 2017	yes	yes			yes/no					yes	yes/no		yes	yes
Reeskens *et al*., 2021					yes/no		yes						yes	
Sawler, 2025				yes		yes/no								yes/no
Senghaas *et al*., 2022a				yes		yes				yes	yes			yes
Senghaas *et al*., 2022b				no						yes	yes/no	yes/no		
Thorp & Larner, 2025	yes/no												yes	
Toossi, 2022	yes/no										yes			
Wang, 2023	yes/no													
Ward & Denney, 2022		yes		yes	yes		yes			yes	yes	no		
Wetts & Willer, 2018	yes/no													
Will, 1993 *							yes	yes		yes	yes/no	yes		yes
Wu, 2021													yes	
Zhirkov *et al*., 2025	yes	no												

*See also Groskind, 1994; Groskind, 1991; Iyengar, 1990; Ponza *et al*., 1988. Note: “Yes” means that all effects are statistically significant. “No” means that none of the effects are significant. “Yes/no” means that some effects are significant while others are not.

This interpretation is supported by the strong and consistent effects associated with three recipient features closely linked to reciprocity: past work history, current employment status, and motivation or effort (typically in terms of seeking a job). Across studies and in line with deservingness theory ( van Oorschot, 2000), framing recipients as having contributed in the past, currently contributing, or willing to contribute in the future reliably elicits more favorable attitudes toward social policies (e.g., Aarøe & Petersen, 2014; Gift & Lastra-Anadon, 2023; Perna & de Reguero, 2025). Similarly, the identified cause of a social risk–whether it is under the recipient's control or not–also has substantial framing effects. For instance, support for generous unemployment benefits typically decreases when recipients are described as having quit their job (e.g., Reeskens & van der Meer, 2018). Likewise, support for public healthcare provision tends to diminish when patients are portrayed as needy due to unhealthy behaviors, such as smoking (e.g., Gollust & Lynch, 2011).

Other recipient characteristics, such as financial need or health status, typically also produce framing effects, although these are often weaker. Generally, depicting policy recipients as needy increases support for social policy (e.g., Baumberg Geiger, 2021; Groskind, 1994). Household composition, particularly the presence of children, tends to positively influence social policy preferences as well (e.g., Will, 1993), though not universally. In some cases–especially when combined with recipients’ ethnic or migration background–these cues may have more complex or even counteracting effects ( Hjorth, 2016; Toossi, 2022). Characteristics such as age and gender yield more inconsistent results, sometimes producing significant effects and at other times not (see Table 3). Additionally, a few recipient features, such as religion and criminal record, have been studied only rarely but tend to show significant effects when examined. Typically, results show that social policy preferences become more negative when recipients are framed as belonging to a minority religion (such as Muslims in Europe; e.g., Ford, 2015) or as having engaged in criminal activities (e.g., Ward & Denney, 2022).

Let us now turn our attention to the second most frequently studied frame content category: policy design. While some studies investigated the framing of social policies’ design more generally (for example, through the provision of a broad information package concerning the overall design of a policy program; e.g., Porumbescu *et al.*, 2025), most zoom in on at least one of the following design dimensions: conditionality, delivery, universality, generosity, and funding (see Table 4 for an overview). The first of these, policy conditionality, refers to both the (usually work-related) obligations and the administrative burdens imposed on those receiving a social benefit or service. Most evidence indicates that citizens’ tend to be more supportive when social policy is purposefully described as including work requirements. For example, it is commonly found that support for a basic income is higher when it is presented as conditional on work or other requirements, compared to a fully unconditional version ( Laenen, 2023; Laenen *et al.*, 2023; Rincón *et al.*, 2022; but see Rincón, 2021). There is, however, less consensus regarding the impact of emphasizing administrative burdens. While some studies suggest that this makes social policy preferences more positive, as it supposedly increases the perceived deservingness of policy recipients (e.g., Keiser & Miller, 2019), others show a negative effect (e.g., Sievert & Bruder, 2024), or generally insignificant effects (e.g., Yang & Hopkins, 2025). Yet others argue that the situation is more complex, with the effects of framing administrative burden differing across social policy programs ( Nicholson-Crotty *et al.*, 2021). Currently, we lack a clear explanation for these contrasting findings, which could be due to differences in factors such as study context (given that the studies were conducted in different countries) and study methodology (e.g., the effects observed in Sievert and Bruder might be due to respondents being asked to imagine that the administrative burdens would be imposed on a *friend* applying for social benefits).

**Table 4.** Overview of policy design features.

	Universality	Conditionality	Generosity	Delivery	Funding
Frequencies (absolute & relative within column) yes no yes/no	4 (28.6%) 1 (7.1%) 9 (64.3%)	7 (43.8%) 4 (25.0%) 5 (31.2%)	5 (55.6%) 1 (11.1%) 3 (33.3%)	4 (40%) 2 (20%) 4 (40%)	5 (38.5%) 1 (7.7%) 7 (53.8%)
Ashok & Huber, 2020				yes	
Baute *et al*., 2022		yes	yes		
Bay & Pedersen, 2006	yes/no				
Bay *et al*., 2016	yes				
Burgoon *et al*., 2022		yes	yes		yes
Campbell & Gaddis, 2017				yes	
Cappelen & Midtbø, 2016	yes				
Carter, 2025					yes
Ellis & Faricy, 2020				yes/no	
Ellis & Faricy, 2021				yes/no	
Fernández *et al*., 2023			yes/no		
Findor *et al*., 2023	yes/no				
Gallego & Marx, 2017	yes/no		yes		yes
Greenberg, 2018	yes/no				yes/no
Haeder & Sylvester, 2024		no		no	yes/no
Haselswerdt & Bartels, 2015				yes	
Haselswerdt, 2022		yes		yes/no	
Jensen & Kevins, 2019			yes/no		
Keiser & Miller, 2019		yes			
Laenen, 2023	yes/no	yes/no	yes/no		yes/no
Laenen *et al*., 2023	yes/no	yes			
Laenen, 2025	yes/no			yes/no	
Lim & Tanaka, 2019	yes				
Muñoz & Pardos-Prado, 2019	yes/no				
Nicholson-Crotty *et al*., 2021		yes/no			
Pedersen, 2019			yes		
Qi & Haselswerdt, 2024			no	no	
Rincón, 2021	yes/no	no			yes/no
Rincón *et al*., 2022	no	yes			yes
Sievert & Bruder, 2024		yes/no			
Stadelmann-Steffen & Dermont, 2020	yes		yes		yes/no
Stenderup & Pedersen, 2025		yes/no			
Yang & Hopkins, 2025		no			

*Note: “Yes” means that all effects are statistically significant. “No” means that none of the effects are significant. “Yes/no” means that some effects are significant while others are not.

Similar contention exists for the dimension of policy delivery, which refers to the way in which social benefits and services are delivered: in kind or in cash, and if the latter, directly via the transfer system or indirectly via the tax system. While several U.S. studies show that preferences are more favorable when social policies are presented as a tax credit rather than a direct cash transfer (e.g., Ashok & Huber, 2020), others report null findings (e.g., Qi & Haselswerdt, 2024). Similarly, whether social policy is presented as being provided in cash or in kind matters according to some studies (with in-kind provision eliciting greater support; Campbell & Gaddis, 2017), but less so in others ( Laenen, 2025).

Also with regard to the design dimension of policy universality, which refers to the eligibility criteria governing access to social benefits and services, we see similar contrasting findings. On the one hand, some studies demonstrate that social policies deliberately described as universally accessible to all citizens/residents tend to receive greater popular support than those described as targeted at the lowest incomes only (e.g., Laenen, 2025; albeit more in Belgium than the US). On the other hand, some studies draw the opposite conclusion, finding that social policies are more popular when these are portrayed as being targeted at the poor only (e.g., Gallego & Marx, 2017). By contrast, highlighting strict citizenship or residency requirements, mainly to exclude newcoming migrants, tends to have a more uniform impact, with support for social policy typically increasing when such requirements are highlighted and decreasing when they are absent. For example, informing citizens about migrants' access to social benefits and services, even within the context of migration within an integrated European Union, strongly decreases support for social spending ( Cappelen & Midtbø, 2016). Importantly, however, there are clear exceptions to this general pattern, notably observed in Spain, where the framing of such requirements does not seem to have a detectable impact on citizens' preferences regarding unemployment benefits ( Gallego & Marx, 2017) or universal basic income ( Rincón, 2021).

There is more unanimity with regard to the dimension of policy generosity, which refers to the level at which social benefits are set. Specifically, studies usually find that framing social policy as being generous tends to increase popular support, at least in the European context, where it has been studied most extensively (e.g., Baute *et al.*, 2022). Finally, the dimension of policy funding, which refers to the way in which social benefits and services are financed, has been studied considerably less. However, when it has been analyzed, it seems to have an impact on social policy preferences. For example, when support for a universal basic income increases when it is described as being financed by progressive income taxation ( Rincón, 2021).

The two other categories of framing content–referring to policy outcomes and policy context–have received much less attention in the literature. Regarding the first, most studies examine frames related to the (anticipated) effects of social policies on economic (e.g., employment rates; Johnson *et al.*, 2023), distributional (e.g., poverty rates; Laenen, 2023), and moral (e.g., benefit abuse; Fang & Huber, 2019) outcome dimensions. Overall, the existing body of evidence suggests that the framing of outcomes tends to affect social policy preferences, with only a few exceptions (e.g., Qi & Haselswerdt, 2024). For example, support for social policy often increases when the policy is described as progressively distributing resources from the rich to the poor, and *vice versa* ( Ashok & Huber, 2020; Guardino & Mettler, 2020; Laenen, 2023). Another pertinent example pertains to framing social policy as costly and damaging to employment or the economy more broadly, which tends to make preferences less favorable ( Haselswerdt & Bartels, 2015; Laenen, 2023; Lim & Tanaka, 2019). There are also a few studies investigating the impact of policy outcomes described at the individual rather than the societal level, usually in terms of the impact on citizens’ income. These generally show that such individualized appeals do affect social policy preferences, with citizens becoming less supportive when they are told they will lose income and more supportive when they think they will gain income, as predicted by self-interest theory ( Jerit, 2009; Laenen & Aerts, forthcoming).

With regard to the content category of policy context, existing studies have investigated the impact of framing three particular types of broader context: the demographic (mostly in terms of population ageing and immigration), economic (mostly in terms of employment rates, fiscal costs, and economic performance), and what I call the “inequality” context, highlighting existing ethnic (e.g., Harell & Liebermann, 2021), educational (e.g., Lergetporer *et al.*, 2020) and financial (e.g., Lindner *et al.*, 2023) inequalities. Overall, it seems that frames related to the economic and inequality context have relatively little impact on social policy preferences, although there are some notable exceptions (e.g., Marx & Schumacher, 2016, who show that support for welfare state retrenchment decreases when rising levels of inequality and unemployment are mentioned). Describing the demographic context, by contrast, tends to have stronger effects on social policy preferences. For example, prior work has shown that pointing out increasing levels of migration negatively affects social policy preferences (e.g., Finseraas *et al.*, 2024). Likewise, emphasizing the ageing of the population has been found to significantly increase acceptance of pension cuts (e.g., Naumann, 2017).

Having examined frame content, I now turn to the characteristics of frame direction, format, level, strength, and frequency, with frame equivalency emerging as a recurring theme throughout the discussion. Let us begin with frame direction, which refers to whether frames are negative–featuring negatively worded messages about policy recipients, design, outcomes, or context–or positive, conveying positively worded messages. As a third option, frames can be more neutral, often serving as the control condition in the experimental studies reviewed here. A classic hypothesis in the literature is that negative frames exert a stronger negative influence than positive frames exert positive effects. This stems from the widely documented tendency of individuals to assign greater weight to negative information than to positive information–a phenomenon known as negativity bias ( Soroka, 2014). But to what extent is this bias observed in empirical research on the framing of social policy preferences? Several studies speak to this question, though their findings point in different directions. Some claim to provide strong support for negativity bias. This is perhaps most evident in Avdagic & Savage (2021), who find that a frame emphasizing the negative consequences of immigration for the welfare state significantly reduced public support for welfare, whereas an equivalent positive frame had a much weaker positive effect (relative to a control group receiving neutral information that did not mention immigration). However, it is worth noting that the positive frame in this study also references growing concerns about the economic impact of immigration, which may have diluted its positivity. Moreover, the same authors ( Avdagic & Savage, 2024) did not find the same negativity bias for welfare chauvinism. Other studies also *appear* to support the hypothesis that negative frames are more impactful than positive ones ( Jordan *et al.*, 2022; Kumlin, 2011; Palmer, 2018), but typically compare frames that are not equivalent–that is, the frames differ not only in direction but also in other respects such as content or strength. This lack of equivalency complicates the interpretation of their findings and renders firm conclusions about negativity bias premature. A notable exception is the study by Laenen (2023), which demonstrates that popular support for a universal basic income declines more sharply when individuals are presented with negative policy outcomes (most notably, rising poverty or reduced employment) than it increases when presented with equivalent positive outcomes (declining poverty or increased employment).

That said, there is also some evidence challenging the notion of a general negativity bias. Most notably, Jerit (2009) finds that a predicted income *loss* did not produce a stronger effect on U.S. citizens’ preferences regarding the privatization of Social Security than an equivalent income *gain* frame. In fact, only the income gain condition differs significantly from the control group (which did not receive any information), which the authors attribute to the role of prior beliefs–specifically, that many respondents already anticipated losses, thus muting the impact of the negative frame. Similarly, Sahn *et al.* (2025) show that a negatively framed message about healthcare access (i.e., ~20% of the population has inadequate access) and an equivalent positive frame (~80% have adequate access) yielded nearly identical (null) effects on people’s opinions about the priority that the U.S. government should give to solving the problem. Taken together, the evidence thus suggests that the role of negativity bias in framing social policy preferences remains unclear and warrants further research using otherwise equivalent frames.

Apart from frame direction, there is remarkably little research on how other frame characteristics influence social policy preferences, despite their importance in the general framing literature. First, only three studies included in this review have investigated the role of frame format by comparing the impact of equivalent frames presented in different formats. Two studies focus specifically on numerical framing, showing that a different presentation of the same numerical information can significantly influence citizens' social policy preferences. For example, citizens seem to prefer higher unemployment benefits when current amounts are presented on a monthly rather than a weekly basis ( Pedersen, 2019; see Pedersen & Larsen, 2019 for a similar “unit effect”, but see McIntyre *et al.*, 2024 for contrasting evidence). From a very different perspective, Porumbescu *et al.* (2025) examined how presenting the same information about the application process and eligibility for the U.S. food stamp program in different formats influences citizens' knowledge and support for the program. They concluded that format matters, as comprehension–and indirectly, support–was higher when respondents were shown an informational video compared to a website or a flyer. Second, among the studies selected for review, only one experiment explicitly studied the strength of frames. Specifically, Callaghan & Olson (2017) found no evidence that strong and weak versions of the same racialization treatment had different effects, as both decreased support for the Earned Income Tax Credit about equally. Other than that, it is often unclear how the frames under consideration differ in terms of their strength, as this is rarely investigated. This is, however, becoming more common in the general framing literature, often assessed through study pretests (e.g., Hopkins & Mummolo, 2017). Third, another relevant characteristic that is seldom studied is the level of frames, which can be either episodic, referring to specific individuals, or thematic, reflecting broader patterns. The only experiment with direct relevance was reported by Kawata *et al.* (2024), showing that episodic framing had a stronger impact than thematic framing. Specifically, they found that citizens who were shown a narrative story about the plight of a single mother were more likely to support higher poverty relief expenditures than those who were shown statistical information about the share of single parents living in poverty in Japan–a phenomenon the authors label as a ‘narrative premium’. However, no such conclusion can be drawn from an inspection of the full body of research, as there is little evidence that framing policy recipients as individuals is generally more effective than framing them as groups (see Table 2).

Finally, the frequency with which frames are communicated is also considered important in the general framing literature but is rarely studied in the context of social policy preferences. While no studies have investigated the impact of repeating the same frame multiple times within a single survey experiment, Berinsky’s (2017) findings are somewhat informative. This study examined how support for the Affordable Care Act (ACA) is influenced by corrections of the rumor that the ACA would install so-called “death panels” deciding over the lives of elderly Americans. In one experiment, respondents were randomly assigned to one of three groups: a treatment group receiving only the rumor, one receiving only the correction, and one receiving both. Subsequently, half of the respondents received a recall question in which the rumor was repeated, while the other half answered an irrelevant question. The results showed that those to whom the rumor was repeated were affected less by the corrective information than those who answered the irrelevant question. This suggests that the effect of frames increases with their frequency. Additionally, several studies demonstrate that in the absence of frame repetition, effects tend to decay over time when the same respondents are surveyed again days, weeks or months after their initial exposure to the frame (e.g., Berinsky, 2017; Finseraas *et al.*, 2017)–a point I will return to when discussing the role of frame context. In sum, although these findings indicate the importance of the format, frequency, level, and strength of frames, more research is clearly needed to assess their role in the framing of social policy preferences.

### The influence of receiver characteristics

As shown in Table 1, most studies included in this review inspect receiver characteristics, by examining the heterogeneity of framing effects across different population subgroups–though some do so more thoroughly than others. This is typically achieved by estimating interactions between treatments and respondent characteristics or by analyzing treatment effects within different sub-samples. To structure the presentation of the main findings, I return to Zaller’s (1992) Receive-Accept-Sample (RAS) model, introduced earlier.

Recall that for framing to occur, the first precondition is that citizens must actually *receive* the frame, which is something they are not all equally capable of or motivated to do. Most studies reviewed here, however, have relatively little to say about situations in which citizens fail to receive a frame, as they typically focus only on those who agreed to participate in a survey and are thus at least somewhat motivated to engage with the frames they are presented with. Still, there are some noteworthy findings related to the “Receive” component of Zaller’s model. First, there is some evidence that framing has no effect on individuals who did not actually receive, or cannot recall receiving, a frame–even if they were supposed to be exposed to it. For instance, Kangas *et al.* (2022) show that sending an information letter about a Finnish pension reform two weeks prior to a survey–designed to assess citizens’ knowledge and attitudes toward the reform–had a significantly stronger effect on respondents who reported actually receiving the letter. Among those who were sent the letter but reported either not receiving it or not remembering it, the impact was negligible. Similarly, Barnes *et al.* (2018) found that sending citizens an informative tax receipt had no discernible impact on their policy preferences, possibly because only 25% recalled receiving it. These findings suggest that survey experiments may often overestimate framing effects, as not all citizens are equally receptive to the information conveyed in real-world settings. Second, even when citizens do receive a frame, their attentiveness to it can vary. This has been more widely acknowledged in the experimental literature, often through the use of attention or manipulation checks. Unfortunately, such checks are frequently used to exclude inattentive respondents altogether (e.g., Toossi, 2022), which again risks inflating observed framing effects by omitting those who, in real-world contexts, would likely pay little attention. Indeed, studies that compare attentive and inattentive respondents typically show that framing effects are substantially stronger among the attentive (see Berinsky, 2017 for an excellent example). Third, even when all citizens receive and attend to a frame, there is likely to be variation in their capacity to understand the information it conveys and to translate that understanding into policy preferences. This is commonly assessed by examining how framing effects vary across individuals with different levels of knowledge–either general (operationalized, for example, through measures of political knowledge, engagement, and interest) or issue-specific (mostly measured through factual questions or self-reported knowledge).

As shown in Table 5, knowledge is one of the few receiver characteristics that consistently moderates framing effects on social policy preferences. A common finding is that more knowledgeable citizens are less susceptible to framing than their less knowledgeable counterparts ( Jäger, 2024; Jensen & Kevins, 2019; Jerit, 2009; Kootstra & Roosma, 2018; Naumann, 2017). For example, Ingham and Levin (2018) found that communicating the results of a deliberative citizen forum on U.S. Social Security reform had markedly weaker effects on the reform preferences of individuals with greater knowledge of, and familiarity with, the policy. Likewise, several studies show that inaccurate prior perceptions increase the likelihood of preference updating in response to new information. For instance, providing correct information about public education spending primarily affected those who initially underestimated it, producing a “sticker shock” effect ( Schueler & West, 2016; see also Lergetporer *et al.*, 2018; Lergetporer *et al.*, 2020). At the same time, however, we should be cautious not to overgeneralize. A few studies find no moderating role for knowledge (e.g., Bendz & Oskarson, 2024; Pedersen & Larsen, 2019), while others even report opposite effects. For example, Bay and Pedersen (2006) found that, contrary to their expectations, attempts to persuade citizens to abandon their initial position toward a universal basic income were *more* successful among the politically interested. At the level of issue-specific knowledge, some studies also find that citizens are actually more responsive to information they are already familiar with, rather than less (see Kawata *et al.*, 2024 for Japan and Song *et al.*, 2024 for Korea).

**Table 5.** Overview of receiver characteristics.

	Racial attitudes	Race/ethnicity	Immigration attitudes	Political ideology/affiliation	Other ideological predispositions *	Deservingness perceptions	Knowledge	Personal experience	Education	Income	Employment status	Household composition	Gender	Age
Frequencies (absolute & relative within column) yes no yes/no	9 (56.2%) 5 (31.3%) 2 (12.5%)	3 (27.3%) 8 (72.7%) 0 (0%)	4 (50.0%) 3 (37.5%) 1 (12.5%	26 (35.6%) 26 (35.6%) 21 (28.8%)	7 (70%) 2 (20%) 1 (10%)	3 (100%) 0 (0%) 0 (0%)	14 (77.8%) 2 (11.1%) 2 (11.1%)	5 (55.6%) 1 (11.1%) 3 (33.3%)	7 (29.2%) 17 (70.8%) 0 (0%)	11 (36.7%) 14 (46.6%) 5 (16.7%)	4 (36.7%) 7 (63.6%) 0 (0%)	3 (30%) 7 (70%) 0 (0%)	5 (38.5%) 8 (61.5%) 0 (0%)	1 (6.7%) 12 (80.0%) 2 (13.3%)
Asticher & Sager, 2025											no	no	no	
Avdagic & Savage, 2021			yes			yes								
Avdagic & Savage, 2024				yes						no	yes			
Barabas *et al.*, 2020				yes										
Baumberg Geiger, 2021				yes										
Baute *et al.*, 2022				yes	yes				yes	yes				
Bay & Pedersen, 2006			yes				yes		no				no	no
Bay *et al.*, 2016			yes											
Bendz & Oskarson, 2022				yes										
Bendz & Oskarson, 2024				no			no							
Berinsky, 2017				yes/no										
Bor & Simonovits, 2021		no		no						no			no	
Bremer & Bürgisser, 2023								yes			yes	yes		
Bridgman *et al.*, 2022										no	no	no		
Buss, 2018				yes										
Callaghan & Olson, 2017	yes/no													
Campbell & Gaddis, 2017				yes										
Cappelen & Midtbø, 2016										no			no	no
Carter, 2025				yes/no										
Cattaneo *et al.*, 2020									no	no			no	no
Chueri *et al.*, 2025				yes/no										
DeSante, 2013	yes													
Doberstein & Smith, 2019				no										
Ellis & Faricy, 2021				yes										
Epp & Jennings, 2020										yes				
Fang & Huber, 2019				yes/no										
Fernández *et al.*, 2023							yes/no							
Finseraas *et al.*, 2024				no										
Ford, 2015	yes			no	no	yes				yes				
Fox *et al.*, 2022				no										
Gallego & Marx, 2017				yes/no					no	no	no			
Gielens *et al.*, 2019					yes									
Gift & Lastra-Anadon, 2023				yes						no	no			no
Gollust & Lynch, 2011		yes												
Greenberg, 2018		yes		yes	yes			yes	yes	yes				
Guardino & Mettler, 2020										yes				
Haeder, 2025a	no	no		no										
Haeder, 2025b	no	no		no										
Haeder *et al.*, 2023	yes/no			yes/no				no	no					
Haeder & Sylvester, 2024	no			no										
Hansen, 2007							yes							
Hansen, 2019				no	yes/no									
Harell *et al.*, 2014	yes													
Harell & Lieberman, 2021	no	no												
Haselswerdt & Bartels, 2015				yes							yes	no		
Haselswerdt, 2020			no											
Haselswerdt, 2022	no		no						no					
Häusermann *et al.*, 2019				yes/no					no	yes/no			yes	
Hjorth, 2016	yes				yes									
Ingham & Levin, 2018							yes	yes						
Jacobsen, 2019				yes/no										
Jacobs & Matthews, 2012									no	yes/no		no	no	no
Jäger, 2024							yes							
Jensen & Kevins, 2019							yes							
Jensen & Petersen, 2017				yes/no							no			
Jerit, 2009							yes							
Johnson *et al.*, 2023				no										no
Jordan *et al.*, 2022				yes										
Kangas *et al.*, 2014				yes/no										
Kangas *et al.*, 2022							yes	yes						
Kawata *et al.*, 2024				no			yes		no	no				
Keiser & Miller, 2019				yes										
Kootstra & Roosma, 2018							yes	yes/no	yes	yes			yes	no
Kuklinski *et al.*, 2000				no			yes							
Kuhn & Kamm, 2019			yes/no	no	no				no					
Kumlin, 2011				yes/no										
Kumlin & Nemčok, 2024				yes/no										
Kweon & Choi, 2022									yes	no	yes			yes
Laenen, 2023				yes/no				yes/no		yes/no			no	yes/no
Laenen *et al.*, 2023				yes/no										
Laenen & Aerts, forthcoming				yes										
Lergetporer *et al.*, 2018		no					yes		no	no	no	no	no	no
Lergetporer *et al.*, 2020				no			yes		no	no		no		
Lim & Tanaka, 2019										yes				
Lindner *et al.*, 2023			no		yes					no				
Magni, 2024				no					no					
Martinangeli & Windsteiger, 2023				no					no	yes/no		no		
Marx & Schumacher, 2016				yes						yes				
McIntyre *et al.*, 2024				yes/no						yes				
Mulayi *et al.*, 2023				yes/no										
Muñoz & Pardos-Prado, 2019			yes			yes			yes					
Myers *et al.*, 2024	yes			yes	yes									
Naumann, 2017				yes			yes							
Naumann, 2018				yes						yes				
Naumann *et al.*, 2024				yes					yes					
Nicholson-Crotty *et al.*, 2021				yes										
Palmer, 2018	yes													
Pedersen & Larsen, 2019				yes/no			no							
Pedersen, 2019				yes/no										
Peffley *et al.*, 1997	yes													
Ponza *et al.*, 1988														yes/no
Porumbescu *et al.*, 2025		no		no						no		no		
Qi & Haselswerdt, 2024				yes/no										
Reeskens *et al.*, 2021				yes										no
Rincón, 2024				yes/no						yes/no	no			
Rodriguez *et al.*, 2010		no		no						no				
Sahn *et al.*, 2025				no										
Schueler & West, 2016							yes/no							
Slothuus & de Vreese, 2010				yes										
Song *et al.*, 2024				yes			yes			yes				
Stadelmann-Steffen & Dermont, 2020				no					no				no	no
Stenderup & Pedersen, 2025				yes/no				yes/no						
Tesler, 2012	yes			no										
Thomas *et al.*, 2023				yes										
Thorp & Larner, 2025				no	yes									
Thorson & Trump, 2024				no				yes						
Toossi, 2022		yes		yes										
van den Heijkant *et al.*, 2024									yes					no
Ward & Denney, 2022				no					no				no	no
Wetts & Willer, 2018		no												
Yang & Hopkins, 2025				yes									yes	
Yeung, 2022				no										
Zhirkov *et al.*, 2025	yes													

*This includes authoritarianism, conservatism, egalitarianism, individualism, and humanitarianism. Note: “Yes” means that all effects are statistically significant. “No” means that none of the effects are significant. “Yes/no” means that some effects are significant while others are not.

Next in Zaller’s (1992) RAS model is the notion that citizens must both accept a frame and sample it when forming opinions. In this regard, it is a long-standing hypothesis that individuals are more likely to accept frames that confirm their prior, often political, predispositions ( Leeper & Slothuus, 2014; Taber & Lodge, 2006). But to what extent is such directional motivated reasoning also evident in the empirical literature on the framing of social policy preferences? While most studies have addressed this by examining the moderating role of political ideology (e.g., conservative vs. liberal views) or partisan affiliation (e.g., Democrats vs. Republicans), others have focused on related ideological predispositions, such as authoritarianism, ethnocentrism, and individualism (see Table 5 for an overview).

One of the most consistent findings in this literature is that citizens who are predisposed to hold negative views toward ethnic minorities and immigrants are especially susceptible to racial or migration-related framing of social policy. These individuals–who also tend to identify more as right-wing voters–typically respond to such frames by adopting more negative social policy preferences. Numerous studies show that framing effects are significantly stronger among these negatively predisposed groups than among more tolerant citizens, who are usually more left-leaning ( Avdagic & Savage, 2021; Avdagic & Savage, 2024; Bay & Pedersen, 2006; Buss, 2018; Callaghan & Olson, 2017; Chueri *et al.*, 2025; Haeder *et al.*, 2023; Hjorth, 2016; Naumann *et al.*, 2024; Palmer, 2018; Peffley *et al.*, 1997; Toossi, 2022; Zhirkov *et al.*, 2025). By contrast, a small number of studies stand out as exceptions, concluding that ideological predispositions do not moderate the effects of racial or migration-related frames. While some of these report null effects across the board (e.g., Harell & Liebermann, 2021), others find that these frames affect individuals similarly, regardless of their ideological orientations (e.g., Magni, 2024).

Beyond frames related to ethnicity, race, and migration, there is substantial evidence that motivated reasoning also occurs in response to other types of framing–particularly when there is a strong alignment between the content of the frame and individuals’ political ideology. Right-wing voters, on the one hand, tend to be more responsive to frames that emphasize issues they traditionally prioritize, such as fiscal costs (e.g., Marx & Schumacher, 2016), benefit conditionality (e.g., Keiser & Miller, 2019), employment effects (e.g., Laenen, 2023), and–in the U.S. context–the level of administration (state vs. federal; e.g., Qi & Haselswerdt, 2024) and mode of delivery (e.g., tax credits vs. cash transfers; e.g., Ellis & Faricy, 2021). Left-wing voters, on the other hand, are typically influenced more by frames related to policy generosity (e.g., Baute *et al.*, 2022) and policy outcomes they strongly support, such as poverty and inequality reduction (e.g., Laenen, 2023).

There is, by contrast, relatively little evidence of backfire or boomerang effects in the reviewed studies, although some exceptions do exist ( Avdagic & Savage, 2024; Kumlin & Nemčok, 2024; Kumlin & Goerres, 2022; Slothuus & de Vreese, 2010). Notably, two of these studies explicitly identify the partisan source of the frame ( Kumlin & Goerres, 2022; Slothuus & de Vreese, 2010), suggesting that partisan cues may contribute to triggering backfire responses (see below). In the remaining studies, the frame sender was not explicitly identified, but it is plausible that participants did infer a partisan source–though this possibility was not directly tested. For instance, Kumlin and Nemčok (2024) find that left-wing respondents became *less* concerned about the sustainability of the welfare state after exposure to a frame emphasizing fiscal threat, which appeared to align with a more right-wing perspective. Finally, it is worth noting that in 36.5% of the studies, political ideology does not moderate framing effects, especially in cases where such heterogeneity was unlikely to begin with (e.g., when the effects of episodic vis-à-vis thematic framing do not vary with partisanship; Kawata *et al.*, 2024).

In addition to ideological predispositions, researchers have (albeit to a lesser extent) also considered the moderating role of citizens’ sociodemographic characteristics, such as their age, gender, and income (see Table 5 for an overview). Compared to ideology, the evidence concerning these sociodemographic moderators, which are typically used as proxies for self-interest, is far more mixed. On the one hand, several studies suggest that citizens respond more strongly to frames that align with their self-interest. For example, Guardino and Mettler (2020) find that low-income earners become significantly less supportive of social tax credit schemes when made aware of their regressive distributional impact, whereas high-income earners are largely unmoved by such framing. On the other hand, many studies report findings that appear to contradict self-interest theory (e.g., Ponza *et al.*, 1988, who report that older respondents were *less* supportive than their younger counterparts of generous social benefits when recipients are framed as being low-income elderly people), or find no evidence that sociodemographic characteristics moderate framing effects (see Table 5). A special case in this regard is ethnic background, which frequently shows no moderating impact on framing effects (e.g., Harell & Liebermann 2021). Nevertheless, there is some evidence suggesting that racial or migration-related frames may either have no effect (e.g., Gollust & Lynch, 2011) or even a reverse effect (e.g., Toossi, 2022) among ethnic minority members–a group that is increasingly significant in most Western societies. It is therefore concerning that so many studies–both American and European–exclude ethnic minorities from their samples when examining the effects of racial or immigration framing, as this practice may introduce systematic bias into the findings ( Callaghan & Olson, 2017; Ford, 2015; Gilens, 1996; Gross & Wronski, 2021; Myers *et al.*, 2024; Palmer, 2018; Reeskens & van der Meer, 2018; Wetts & Willer, 2018).

This leads to a final, critical point about the influence of receiver characteristics: the frequent failure to test for heterogeneity in treatment effects. Much of the literature on the framing of social policy preferences reports only average treatment effects across the full sample (i.e., 42 studies in this review), thereby obscuring important subgroup variation. This omission carries two main risks. First, it may create the false impression that a framing effect is universal when it actually applies only to specific subgroups. Racial framing effects, for instance, are often limited to individuals with particularly negative priors toward ethnic minorities and migrants, such as radical right-wing voters (see above). Second, average treatment effects can lead to misleading conclusions when opposing subgroup responses cancel each other out. For instance, some studies (e.g., Avdagic & Savage, 2024) show that left-wing and right-wing voters sometimes move in opposite directions in response to the same frame, which could lead to the erroneous conclusion that the frame is ineffective if subgroup dynamics are not taken into consideration.

### The influence of sender characteristics

In contrast to the extensive attention paid to the characteristics of frames and their receivers, surprisingly little focus has been given to the characteristics of those sending the frames in the literature on social policy preferences. This is particularly noteworthy given the widely held assumption that “the messenger may matter more than the message itself”, as well as the growing body of evidence in the general framing literature indicating that the source of a frame can significantly influence its impact (e.g., Druckman, 2001). Often, the studies included in this review leave the frame sender unspecified, prompting respondents to infer that the source is the entity administering the survey–typically indicated at the beginning of the questionnaire. When a frame source is specified, it is typically attributed either to political actors–such as government agencies (e.g., Pedersen & Larsen, 2019), politicians (e.g., Berinsky, 2017), or interest groups (e.g., van den Heijkant *et al.*, 2024)–or to other sources, including research institutes (e.g., Laenen & Aerts, forthcoming) and news media (e.g., Baumberg Geiger, 2025). Importantly, however, only 5 of the 163 studies included in this review have explicitly varied the frame sender, allowing for a direct assessment of its impact (see Table 1). These studies typically find that source cues matter, but mostly in interaction with characteristics of the frame receivers. For example, in their analysis of citizens’ support for a policy proposal to privatize in-home care for senior citizens in Denmark, Slothuus and de Vreese (2010) conclude that information about the political party making the proposal (either the left-wing Social Democratic Party or the right-wing Liberal Party) matters only in that Liberal voters become more supportive of the proposal when it is sponsored by their own party. Among the most politically aware Liberal voters, a backfire effect was even observed: when the policy was framed by the Social Democratic Party in terms of the disadvantages of privatization, these voters became *more* rather than less supportive of it. No such effects were found among Social Democratic voters (but see Kumlin, 2011 and Kumlin & Goerres, 2022 for similar findings demonstrating partisan motivated reasoning among left-wing voters). In a similar vein, Tesler (2012) demonstrates that attributing the Affordable Care Act to either Barack Obama or Bill Clinton had no effect in the full sample, but did increase opposition to public healthcare among respondents with high levels of racial resentment. Similarly, Jacobsen (2019) finds that Americans’ opinions on health, housing, and labour policies are only influenced by information about these policies’ cost-effectiveness when it comes from “their” partisan source rather than from the opposing political camp. Importantly, however, the information had an even stronger impact when delivered by a more neutral, non-partisan research organization, particularly among moderates lacking a strong partisan affiliation.

Beyond these examples, the evidence for messenger effects in the literature on the framing of social policy preferences remains largely circumstantial. A case in point is Yeung (2022), who suggests that U.S. conservatives’ continued opposition to a universal basic income–even when presented with ideologically resonant arguments (e.g., emphasizing its potential to reduce government bureaucracy)–may stem from the fact that a large majority of them (75%) strongly associated the policy with the Democratic Party. However, like much of the existing literature, the role of the frame source was not directly investigated, leaving the influence of sender characteristics largely inferential. Moreover, the limited evidence currently available focuses almost exclusively on senders’ political affiliation, offering little insight into other potentially relevant characteristics (but see Tesler, 2012 on the role of race). A further exception is van den Heijkant *et al.* (2024), who find that the media source of a frame–whether presented in a printed newspaper or on Facebook–had no discernable impact on citizens’ pension preferences, even among respondents who received the frame through their preferred news medium. Overall, however, our understanding of the broader range of sender characteristics that may shape framing effects on social policy preferences is extremely limited.

### The influence of context characteristics

Especially compared to receiver characteristics, the literature on social policy preferences has paid considerably less attention to another contingency factor frequently emphasized by framing scholars: the broader context in which frames are communicated (e.g., Chong & Druckman, 2007). This is not to say that the role of context has been wholly disregarded. In fact, many of the studies included in this review invoke contextual factors either to develop theoretical hypotheses regarding the presence or absence of framing effects, or to explain empirical findings post hoc. A recurring focus within these discussions is the existing information environment, particularly the matter of issue salience. Many studies explicitly select policy cases based on their presumed public or political salience, often targeting issues deemed especially salient at the time of the survey. However, this selection strategy carries the risk of inducing pretreatment bias ( Druckman & Leeper, 2012), as citizens are more likely to have prior exposure to, and knowledge of, highly salient issues. For example, the study by Tesler (2012) introduced above found no differences between the Obama condition and the control group, which did not receive any information about the frame source, leading the author to conclude that most Americans probably already had Obama in mind when forming opinions about the Affordable Care Act. Beyond the information environment, other contextual factors are also considered, including macroeconomic conditions (e.g., Gallego & Marx, 2017), welfare state institutions (e.g., Reeskens & van der Meer, 2017), and shock events like the Covid-19 pandemic (e.g., Haeder *et al.*, 2023). For example, the finding that Spanish citizens are more supportive of social benefits when they are described as generous has been repeatedly attributed to the country’s comparatively high unemployment rate (e.g., Rincón, 2021).

Although context is thus widely recognized as a relevant moderator of framing effects, direct empirical tests of its influence remain relatively limited in the literature on social policy preferences. Most studies included in this review were conducted at a single time and place, precluding the examination of contextual variation. Nonetheless, there are exceptions (32 studies included in this review, to be exact), which can be grouped into three categories. The first and largest category includes studies that conduct survey experiments across different (mostly European) countries to examine cross-national variation in framing effects. The findings here are mixed. On the one hand, some studies report highly similar framing effects across national contexts. For example, Aarøe and Petersen (2014) show that Americans and Danes, despite holding divergent stereotypes about social policy recipients, respond similarly to frames signaling low job-seeking efforts: people in both countries perceive such recipients as significantly less deserving of social support. On the other hand, other studies identify important cross-national divergences. Goerres *et al.* (2020), for example, find that frames highlighting reform pressures on the welfare state influence perceptions of sustainability in Norway and Sweden, but not in Germany. Most studies, however, report both cross-national differences and similarities, depending on the specific frames and outcome variables. Avdagic and Savage (2021), for instance, show that while citizens in Germany, Sweden, and the UK all respond negatively to information about migration’s burden on the welfare state, only Swedes also respond positively to information portraying migration as beneficial. The second category leverages regional variation within countries, typically showing that framing effects vary across regions depending on their demographic, economic, or political situations ( Finseraas *et al.*, 2024; Laenen, 2023; McIntyre *et al.*, 2024; Schuetz *et al.*, 2023; van der Meer & Reeskens, 2021). For example, Haselswerdt (2020) finds that U.S. citizens living in neighborhoods with larger Hispanic populations are more likely to view social assistance as benefiting migrants when exposed to cultural and fiscal threat frames, compared to those residing in more homogeneous areas.

The third category explores the temporal rather than geographical dimension of context, examining how framing effects evolve over time. This is done either by repeating experiments among (a subsample of) the same respondents (e.g., Finseraas *et al.*, 2017), or by conducting follow-up surveys with different samples (e.g., Gramacho *et al.*, 2025). Also here, the evidence points in different directions. While some studies suggest that framing effects are relatively stable over time ( Greenberg, 2018; Häusermann *et al.*, 2019; Kuziemko *et al.*, 2015), others report–often rapid and strong–decay in these effects ( Berinsky, 2017; Finseraas *et al.*, 2017; Jäger, 2024). Still others adopt a more nuanced stance on the role of time. Most notably, Gramacho *et al.* (2025) repeated the same experiment at multiple points in time, to test whether the observed variation in media and political attention for the issue at hand–a proposed reform of Brazil’s social security system–had an impact on how framing affected public opinion about that reform. Their results show that framing significantly shaped reform preferences only when issue salience was low. Conversely, when the reform was highly salient, framing effects disappeared, which highlights the moderating role of salience and the potential for pretreatment bias when issues feature prominently in public discourse at the time of surveying.

In sum, although the role of both temporal and geographic context in shaping framing effects on social policy preferences is increasingly acknowledged, the empirical evidence remains inconclusive, underscoring the need for further research on the matter.

## A conceptual framework and research agenda

This scoping review of more than 160 published experimental studies unmistakenly demonstrates that the extreme positions–claiming that framing effects on social policy preferences are either inherently strong or inherently weak–are misguided. Instead, the evidence generally supports the view, now dominant in the general framing literature, that these effects are highly contingent on a set of characteristics related to (1) the frames themselves, (2) the receivers of those frames, (3) the senders of the frames, and (4) the context in which frames are communicated. A telling example concerns the framing of social policy as primarily benefiting–or even being threatened by–ethnic minorities or immigrants. While such frames are often assumed to uniformly reduce support for social policy, the empirical evidence reveals a far more nuanced reality. Frequently, racial or migration-related frames have no measurable impact at all. And when effects do emerge, they are typically moderated by various factors, including characteristics of the frames themselves (e.g., their direction; Avdagic & Savage, 2021), the receivers (e.g., levels of racial resentment; Peffley *et al.*, 1997), the senders (e.g., political affiliation; Kumlin & Goerres, 2022), and the broader context (e.g., ethnic composition of the local community; van der Meer & Reeskens, 2021). At the same, the review finds that, while empirical support exists for each of these factors–albeit more for some than for others–important knowledge gaps remain, highlighting clear priorities for future research.

First, while frame characteristics have received considerable (albeit often implicit) attention in the literature on social policy preferences, there is a pressing need for more direct and systematic investigation of their impact–especially of frames’ direction, format, frequency, level, and strength. For one, we need more research directly comparing the effects of episodic versus thematic framing. Similarly, future work should more carefully assess frame strength, which could be evaluated in pretests and subsequently manipulated in the main experiments. The frequency with which citizens encounter frames is another important but underexplored dimension, which could be examined by exposing the same respondents to identical or highly similar frames multiple times, either within a single survey or in successive waves. Future research should also disentangle the effects of frame direction, ideally by comparing frames that are identical in all respects except for their valence, to rigorously test for negativity bias. A similar principle applies to frame format: future research wanting to isolate its effect will have to ensure that the frames they investigate are equivalent across all other dimensions.

Second, greater attention should be directed toward sender characteristics, which remain underexplored compared to receiver characteristics in the literature on the framing of social policy preferences. Although the limited existing evidence indicates that source cues typically do influence framing effects, most research has narrowly focused on party-based cues. Future studies should broaden the scope of sender characteristics under investigation–such as whether a frame is attributed to a political party or a research institute (see Jacobsen, 2019)–to deepen our understanding of how messenger effects shape the framing of social policy preferences.

Third, future research should more thoroughly examine the moderating role of the broader context in which frames are communicated, both geographically and temporally. This includes studying framing effects on social policy preferences across different countries or regions, as well as over time. Ideally, such investigations should be guided by theoretically grounded hypotheses about specific contextual moderators–such as issue salience (see Gramacho *et al.*, 2025)–rather than by arbitrary case selection. Moreover, there is an urgent need for greater contextual variation, especially through studies conducted beyond Europe and the US. Another important avenue for future research is a thorough investigation of the persistence of framing effects on social policy preferences over time. Additionally, how these preferences take shape in contexts featuring competing frames also remains underexplored in current research, warranting further scholarly attention (see Hansen, 2007 for an exception).

Finally, perhaps the most pressing recommendation for future research is to systematically explore *interactions* among the four contingency factors identified in this review. A few studies already show that such interactions matter: for example, when partisan source cues often only affect attitudes when aligned or misaligned with the receiver’s political affiliation (see Kumlin, 2011). Many other interactions are theoretically plausible, suggesting that framing effects emerge from a complex interplay between the characteristics of frames, receivers, senders and context. These interactions could involve two factors–as noted by some previous studies–or extend to more complex, higher-order interactions involving three or even all four contingency factors. For instance, the effect of a migration-related frame on citizens’ social policy preferences may depend on the specific combination of multiple factors, including the format in which the frame is conveyed (e.g., episodic vs. thematic), its sender (e.g., a research institute vs. political party), the political beliefs of the receiver (e.g., right-wing vs. left-wing) and the broader demographic context (e.g., heterogeneous vs. homogeneous).

**Figure 1.**
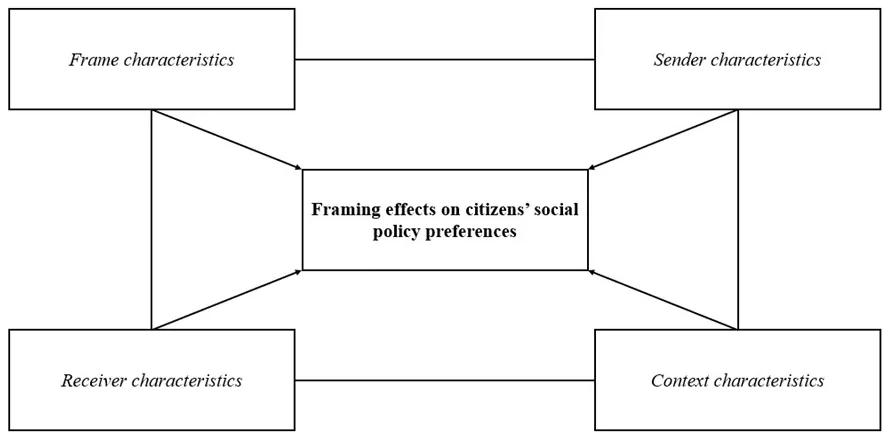
A conceptual framework for future research.

To navigate this complexity, Figure 1 presents a novel conceptual framework designed to inspire and guide future research on the framing of social policy preferences. At its core, the framework proposes that framing effects are conditional on the complex interplay between four sets of contingency factors: frame characteristics, receiver attributes, sender features, and contextual conditions. By integrating dimensions that, although recognized in prior research, have rarely been put together in such a structured way, the framework systematizes and extends existing theories of contingent framing. In doing so, it also provides a fertile foundation for developing new theoretical hypotheses to clarify the mechanisms through which framing affects policy preferences. Importantly, although developed here specifically for social policy, the framework also has broader relevance, as it is transferable to other key policy domains–such as climate, defense, or immigration–where similar framing dynamics could occur.

Methodologically, I strongly recommend the use of multi-dimensional survey experiments that simultaneously manipulate multiple contingency factors as a particularly promising strategy to advance this research agenda. To the best of my knowledge, no existing study has employed such a design, despite the clear signals that framing effects are shaped by interactions between frames, senders, receivers, and context. Implementing such experiments would represent a significant methodological innovation, enabling researchers to capture the complex, conditional nature of framing effects on (social) policy preferences. For example, a factorial vignette experiment could assess both the separate and combined effects of factors such as frame direction, format, source, and context within a single design. Naturally, not all contingency factors lend themselves equally well to such experimental manipulation. Some are better assessed through observational measures, including many receiver characteristics–such as citizens’ political affiliations–as well as certain sender and context attributes, like perceived source credibility or the real-world salience of a policy issue. It is only by combining high-quality observational data with sophisticated factorial experiments that we can begin to rigorously test theoretically informed hypotheses about the interplay among frame, receiver, sender and context characteristics in shaping framing effects on (social) policy preferences.

## Ethics and consent

*Ethical approval and consent were not required*

## Data Availability

No data are associated with this article.
