# Supplementary material for: Framing social policy preferences: A scoping review and research agenda
Source: Open Res Eur. 2026 Aug 20;5:371. Originally published 2025 Dec 1. [Version 2] doi: 10.12688/openreseurope.21596.2 (PMC13562995; doi:10.12688/openreseurope.21596.2)
Supplement: Peer review history

# Reviewer report: Reviewer response for version 2

**DOI:** 10.21956/openreseurope.26486.r79586

**Published:** 2026-10-08

**Authors:** Boglárka Herke

**Affiliations:** 1 ELTE Centre for Social Sciences, Budapest, Hungary

I agree with the previous reviewers that this article is an important contribution to the literature, particularly because it provides a comprehensive review of framing effects.  It is also very ambitious in its coverage of a substantial body of research, with 160 studies included in the review. I also see it as a strength that the paper moves beyond simply reviewing the existing studies and, instead, uses the review to develop a new research agenda.

 I agree with the previous reviewers that the article uses the concept of “framing” in a rather broad sense. However, given the author’s response and the additional information provided in the revised manuscript, the rationale for adopting this broad conceptualisation is now much clearer.

 That said, I think it would still be useful to reflect somewhat more explicitly on the selection of studies in the methodological section. At present, only some of the key search terms are listed (e.g., framing, priming, information provision, persuasion). For the sake of transparency and reproducibility, I would recommend providing the complete list of search terms, perhaps in an appendix rather than in the main text.

 It would also be useful to provide more information on the inclusion of different experimental designs. While the characteristics of the designs are presented on p. 7 in the overview of the studies, I miss a description of the selection process and, in particular, the rationale for including different experimental designs. While I think it is now clear at the theoretical level how the paper conceptualises framing, it would be helpful for readers if the methodology section specified the selection criteria in more detail. In particular, was the presence of a control group without any framing manipulation required for a study to be classified as examining framing effects?  Or, in the case of survey experiments, was the comparison of multiple experimental conditions considered sufficient to identify framing effects by comparing different framing conditions with one another? Clarifying this criterion would help readers better understand how studies were selected and how “framing effects” were operationalised in the review.

 While information on the publication years of the included studies has now been added to the revised manuscript, it would still be useful to clarify this aspect in the methodological section. Specifically, was a predefined time frame used when searching for relevant articles, or was no temporal restriction imposed? If no predefined time frame was used, a brief statement clarifying this would be sufficient.

 I would encourage the author to provide some further reflection on the inclusion of unpublished or forthcoming manuscripts, such as Leanen and Aerts (forthcoming). In particular, it would be helpful to clarify whether such studies had already undergone peer review at the time of inclusion and what criteria were used for including unpublished work. This would further strengthen the transparency of the inclusion process.

 Finally, in relation to the last chapter and the future research agenda, I would recommend reflecting on the potential role of control groups when testing different framing characteristics. In particular, it would be useful to consider how the inclusion of a control condition, in which respondents receive no specific framing, could help assess how opinions differ across various framing conditions and disentangle the effects of different framing strategies. More broadly, it may be worth considering not only how different framing strategies affect opinions relative to one another, but also how they compare to a condition in which no specific framing is provided.

Are all factual statements correct and adequately supported by citations?

Yes

Is the review written in accessible language?

Yes

Are the conclusions drawn appropriate in the context of the current research literature?

Yes

Is the topic of the review discussed comprehensively in the context of the current literature?

Yes

Reviewer Expertise:

welfare attitudes, deservingness, survey experiments

I confirm that I have read this submission and believe that I have an appropriate level of expertise to confirm that it is of an acceptable scientific standard, however I have significant reservations, as outlined above.

# Reviewer report: Reviewer response for version 2

**DOI:** 10.21956/openreseurope.26486.r79588

**Published:** 2026-10-08

**Authors:** Tamara Popic

**Affiliations:** 1 Sant'Anna School of Advanced Studies, Pisa, Tuscany, Italy

This article provides a systematic scoping review of more than 160 studies that use survey experiments to examine how framing affects citizens' social policy preferences. The author assesses three theoretical perspectives in the literature: one that sees social policy preferences as highly malleable, one that stresses their stability because they are rooted in ideological predispositions and self-interest, and a third that sees framing effects as contingent on several factors. The review finds strong support for the contingency view. Framing effects on social policy preferences are neither inherently strong nor inherently weak; they depend on characteristics of the frame, the receiver, the sender and the context in which frames are communicated. The review also shows that existing research has concentrated mainly on receiver characteristics, usually by estimating treatment heterogeneity across ideological and sociodemographic subgroups. Much less is known about sender characteristics, frame characteristics (direction, format, frequency, level and strength) and the broader context. Above all, few studies have examined how these contingency factors interact. On this basis, the author proposes a research agenda and a conceptual framework (Figure 6) that calls for studying these factors in combination rather than in isolation.

 The article makes a very valuable contribution to the field. It is a much-needed systematic review of a large and fast-growing but fragmented body of experimental research, and it is necessary for setting future avenues of research on welfare attitudes, and on welfare preferences in particular. The proposed research agenda is ambitious, and its emphasis on the interactions between contingency factors promises significant gains in our understanding of how framing shapes social policy preferences in real-world settings. The review is very clearly written, comprehensive in scope and well organised around the four contingency factors. I have one major comment and one smaller one, which I hope will help the author strengthen the article further.

 My main concern is that the review treats social policy, and social policy preferences, largely as a whole. The review covers a wide range of policy domains, including unemployment benefits, healthcare, old-age pensions, disability and sickness benefits, childcare, social assistance, housing and education. However, the analysis and the research agenda rarely consider how framing effects might vary across these welfare sectors. Several findings reported in the review are in fact limited to specific types of social policy. For example, the effects of work requirements and reciprocity-related recipient features apply mainly to unemployment benefits and social assistance, while the effects of portraying patients as responsible for their need through unhealthy behaviour apply to healthcare. Treating welfare preferences as a whole risks losing sensitivity to these differences.

 This matters especially for the contextual dimension of the framework. As currently developed, the context dimension does not consider policy-specific contexts or policy-specific welfare preferences. The author partly addresses this by stressing the need to take issue salience into account. However, more structural differences between social policy fields should also be considered, such as differences in types of risks they cover, in institutional design, in who benefits and who pays, and in how directly citizens are exposed to a given policy.

 Relatedly, the conclusion stresses that the proposed framework is useful for research on preferences in other, non-welfare policy fields such as climate, immigration and defence. I would suggest that the author also reflect on the possible limitations of the framework, or on the relative importance of its elements, within welfare sector, i.e. across different welfare policy fields. For example, should we expect receiver characteristics to matter equally in the context of preferences for healthcare and in the context of preferences for unemployment policy? Healthcare is a life-course risk, and near-universal service that almost all citizens use at some point, whereas unemployment benefits relate to labor market risks, and target a narrower and often stigmatised reciever group. This may shape both how receptive citizens are to frames and which receiver characteristics moderate framing effects.

 Similarly, the multi-dimensional survey experiments that simultaneously manipulate several contingency factors, such as the factorial vignette design proposed in the research agenda, are a very promising avenue for future research. In discussing them, however, it would be important to also reflect on the possible variation of framing effects on preferences across different welfare sectors. Policy field could itself be treated as a dimension to vary, or at least as a condition under which the relative weight of the four contingency factors may change. \

 I also have a more technical comment, on the placement of Figure 1. Figure 1 currently appears on page 6, before the section in which it is discussed. It should be moved into the Results section close to its in-text reference.

Are all factual statements correct and adequately supported by citations?

Yes

Is the review written in accessible language?

Yes

Are the conclusions drawn appropriate in the context of the current research literature?

Partly

Is the topic of the review discussed comprehensively in the context of the current literature?

Yes

Reviewer Expertise:

Welfare attitudes, Welfare policy and politics

I confirm that I have read this submission and believe that I have an appropriate level of expertise to confirm that it is of an acceptable scientific standard, however I have significant reservations, as outlined above.

# Reviewer report: Reviewer response for version 2

**DOI:** 10.21956/openreseurope.26486.r79457

**Published:** 2026-09-09

**Authors:** Isabelle Stadelmann-Steffen

**Affiliations:** 1 University of Bern, Bern, Switzerland

The author has considered and integrated my comments in a satisfactory way. I do not have further comments.

Are all factual statements correct and adequately supported by citations?

Yes

Is the review written in accessible language?

Partly

Are the conclusions drawn appropriate in the context of the current research literature?

Partly

Is the topic of the review discussed comprehensively in the context of the current literature?

Yes

Reviewer Expertise:

Public opinion, policy preferences, survey embedded experiments, welfare state and energy policy

I confirm that I have read this submission and believe that I have an appropriate level of expertise to confirm that it is of an acceptable scientific standard.

# Reviewer report: Reviewer response for version 2

**DOI:** 10.21956/openreseurope.26486.r79458

**Published:** 2026-08-31

**Authors:** Marius R Busemeyer

**Affiliations:** 1 University of Konstanz, Konstanz, Germany

The author has responded in a very comprehensive and convincing manner to the points raised in the previous review. In my view, this article now represents a very important and useful overview of the literature on framing and social policy preferences, which is likely to be read a lot.

Are all factual statements correct and adequately supported by citations?

Yes

Is the review written in accessible language?

Partly

Are the conclusions drawn appropriate in the context of the current research literature?

Yes

Is the topic of the review discussed comprehensively in the context of the current literature?

Yes

Reviewer Expertise:

Comparative welfare state research, comparative political economy, public attitudes towards the welfare state, political and socio-economic inequality

I confirm that I have read this submission and believe that I have an appropriate level of expertise to confirm that it is of an acceptable scientific standard.

# Reviewer report: Reviewer response for version 2

**DOI:** 10.21956/openreseurope.26486.r79581

**Published:** 2026-08-28

**Authors:** Arlo Poletti

**Affiliations:** 1 University of Trento, Trento, Italy

This article provides an ambitious and potentially valuable synthesis of a large and fragmented literature on how communicative interventions affect social-policy preferences. The revised version has improved in several respects. In particular, the connections with policy-feedback and deservingness research are now more explicit; the heterogeneity of the dependent variables is acknowledged more clearly; and the new figures and subsections make the article considerably easier to navigate.

 Nevertheless, I retain one important conceptual reservation. Both previous reviewers raised fundamental concerns regarding whether all the studies included in the review can appropriately be classified as studies of framing, especially when experiments manipulate substantive policy-design features or characteristics of policy recipients. The revised manuscript responds by defining framing as the “selective communication of policy-relevant information.” It argues that even experiments concerning objective policy attributes necessarily select which information is presented, which aspects are emphasized, and how they are formulated. This clarification makes the author’s position more explicit. However, I wonder whether it suffices to fully resolve the underlying conceptual tension identified in the previous version of the article. True, all experimental treatments necessarily select and communicate some information while omitting other information. However, there remains the question whether, being a general feature of communication, selectivity can provide a sufficient criterion for identifying framing by itself: if framing is defined in this broad manner, virtually every survey experiment concerning policy preferences becomes a framing experiment. This risks making the concept analytically non-discriminating and obscuring potentially consequential differences among the causal processes examined in the reviewed studies.

 I see two viable ways of addressing this concern. The first would be to retain framing as the article’s overarching concept while clarifying its boundaries vis-à-vis other informational interventions. This would require distinguishing more systematically among at least three types of treatment. Framing changes the presentation, emphasis, or interpretive organization of a substantially equivalent policy object—for example, describing the same outcome in terms of gains rather than losses. Information provision changes the factual or argumentative inputs available to respondents—for example, by informing them about the fiscal cost or distributive consequences of a program. Policy- or recipient-attribute manipulations change the substantive object being evaluated—for example, by comparing programs with different eligibility conditions or recipients with different characteristics. In the latter case, respondents may simply prefer one policy design or beneficiary over another, which does not necessarily constitute a framing effect. The boundaries among these categories are admittedly porous. An informational treatment may simultaneously emphasize one consideration, while an attribute experiment may contain a framing component. The studies therefore need not be placed in mutually exclusive categories. They could instead be coded according to whether the intervention adds information, alters the presentation of equivalent information, changes the salience of particular considerations, or modifies the substantive policy or recipient. Such a typology would allow the author to retain the broad empirical corpus while avoiding the implication that all selective communication is necessarily framing.

 The second option entail repositioning the article itself as a review of “communicative interventions” affecting social-policy preferences. Framing, priming, persuasion, information provision, and policy- or recipient-attribute manipulations could then be treated as related but analytically distinguishable forms of communicative intervention. This may, in fact, better reflect the actual scope and distinctive contribution of the article. Its principal achievement is not limited to synthesizing a narrowly defined framing literature; rather, it brings together bodies of work that use different concepts and experimental designs to study how the communication of policy-relevant considerations affects citizens’ preferences. Adopting this broader conceptual umbrella would come with the advantage of preserving the inclusiveness of the review without stretching the meaning of framing beyond its analytically useful limits.

 Either option would also help qualify the article’s main conclusion. The reported variation in findings may reflect genuine contingency in the effects of communicative interventions, but it may also result from combining interventions that operate through different mechanisms and dependent variables that capture different outcomes. For this reason, I would recommend replacing claims that the evidence “unmistakenly demonstrates” the contingency perspective with the more cautious conclusion that the evidence is broadly consistent with it. Given the conceptual ambiguities discussed, it remains dubious whether the review can conclusively determine how much of the observed variation reflects causal moderation and how much derives from differences in treatments, outcomes, samples, and research designs.

 Overall, this is a useful and impressively wide-ranging review, and the revised version represents meaningful progress. However, because the conceptual choice determines both the composition of the reviewed literature and the interpretation of its findings, this is a substantive rather than merely terminological concern. I do not have a strong preference between the two options outlined above. It seems to me that both could provide the article with a more coherent conceptual foundation.

Are all factual statements correct and adequately supported by citations?

Yes

Is the review written in accessible language?

Yes

Are the conclusions drawn appropriate in the context of the current research literature?

Partly

Is the topic of the review discussed comprehensively in the context of the current literature?

Yes

Reviewer Expertise:

Political Science, International Political Economy, International Relations,

I confirm that I have read this submission and believe that I have an appropriate level of expertise to confirm that it is of an acceptable scientific standard, however I have significant reservations, as outlined above.

# Response

**Published:** 2026-09-11

**Authors:** Tijs Laenen

**Affiliations:** Sociology, Antwerp University Faculty of Political and Social Sciences, Antwerp, Flanders, Belgium

Thank you for this thoughtful and constructive review. The review raises an important question concerning the boundaries of framing and its relationship to information provision and policy- or recipient-attribute manipulations. I agree that the distinctions outlined by the reviewer are theoretically meaningful and may prove useful for future work. At the same time, I remain persuaded by the broader conceptualization adopted in the review. In my view, the studies included share a common focus on how the communication of policy-relevant information shapes social policy preferences. While they differ in the specific characteristics they manipulate, they all involve presenting respondents with selectively chosen information about policies or policy beneficiaries. For that reason, I consider them part of the broader body of research that the review seeks to synthesize. The typology proposed by the reviewer offers a valuable alternative perspective on how this literature might be organized. More generally, I see the reviewer's comments as highlighting an important ongoing debate regarding the conceptual boundaries of framing and related communicative interventions. While I appreciate the alternative perspective offered by the reviewer, I remain persuaded by the broader conceptualization adopted in the review. I would like to thank the reviewer once again for their careful reading of the manuscript and for their valuable contribution to this discussion.

# Reviewer report: Reviewer response for version 1

**DOI:** 10.21956/openreseurope.23364.r71090

**Published:** 2026-04-07

**Authors:** Isabelle Stadelmann-Steffen

**Affiliations:** 1 University of Bern, Bern, Switzerland

This paper is very ambitious. It presents a scoping review based on over 160 articles, condensing the main findings related to the question of how 'framing' affects social policy preferences. Its main merit lies in the identification and coding of these articles, and the dense summary of the relevant findings of this literature.

 Nevertheless, I have a few concerns and suggestions.

 Firstly, is this really all about 'framing'? While I understand why the author conceptualises 'framing' so broadly, I have some concerns that this broad conceptualisation might be too broad to be analytically useful. In particular, when it comes to the content of the framing and policy design, which seems often a main focus of factorial experiments, I wonder whether this can really be considered 'framing'. In my understanding, most of these studies would not consider themselves as «framing studies» but more likely link to «policy design» or even policy feedback. Does considering these studies as studies about 'framing' not actually lead to a misinterpretation of the role of framing in policy preferences?

 Secondly, not only is 'framing' understood very broadly, but 'social policy preferences' also means very different things in different studies. As a result, the review merges studies with very different dependent variables. This is not a bad thing as such. However, to make this heterogeneity more transparent, I suggest that the tables presenting the studies should contain information about the dependent variable and the experimental approach. This would enable readers to see more clearly what the different studies are about and draw their own conclusions. Furthermore, I believe this variety should be given more consideration when interpreting the results.

 Thirdly, and relatedly, I wonder if the author could explore whether the 'conditionalities' are systematically linked to different types of framing or dependent variables. The main conclusion that it is all about conditionalities seems realistic. However, to make this claim more pertinent and relevant, it would be important to discuss whether the many conditional and 'inconclusive' findings are really the result of conditionalities or, to a certain extent, simply the result of examining articles that ultimately study very different things. I can see two ways forward: Either the author should demonstrate more clearly that the identified conditionalities are relevant beyond specific conceptualisations of 'framing' and different dependent variables, or the author should attempt to systematise these conditionalities in relation to types of framing and/or dependent variables.

**Minor points:**

- What is really helpful is that specific studies are mentioned in the text to underline one or the other argument. However, the selection of these illustrative studies is somewhat unbalanced. While some studies are referenced repeatedly, others that would also fit are never mentioned.
- At several instances in the paper, formulations such as 'more recently' are used to refer to quite references that are actually quite old (or not recent, in any case).
- It is not clear what time period the scoping review covers. Why are the starting and end points not defined? Some of the studies are very old, which could mean that 'outdated' findings are influencing the review's results.
- The tables are important, but they are also very long and difficult to read. Perhaps consider including summary tables in the main text and moving the long tables to the appendix.
- The criticism that many studies do not examine subgroup differences seems to be phrased rather harshly. There may be reasons why subgroup differences are not relevant or have not been studied. Alternatively, they may have been studied, but no heterogeneity was found.

Are all factual statements correct and adequately supported by citations?

Yes

Is the review written in accessible language?

Partly

Are the conclusions drawn appropriate in the context of the current research literature?

Partly

Is the topic of the review discussed comprehensively in the context of the current literature?

Yes

Reviewer Expertise:

Public opinion, policy preferences, survey embedded experiments, welfare state and energy policy

I confirm that I have read this submission and believe that I have an appropriate level of expertise to confirm that it is of an acceptable scientific standard, however I have significant reservations, as outlined above.

# Response

**Published:** 2026-07-08

**Authors:** Tijs Laenen

**Affiliations:** Sociology, Antwerp University Faculty of Political and Social Sciences, Antwerp, Flanders, Belgium

**This paper is very ambitious. It presents a scoping review based on over 160 articles, condensing the main findings related to the question of how 'framing' affects social policy preferences. Its main merit lies in the identification and coding of these articles, and the dense summary of the relevant findings of this literature.**

**Nevertheless, I have a few concerns and suggestions.**

**Firstly, is this really all about 'framing'? While I understand why the author conceptualises 'framing' so broadly, I have some concerns that this broad conceptualisation might be too broad to be analytically useful. In particular, when it comes to the content of the framing and policy design, which seems often a main focus of factorial experiments, I wonder whether this can really be considered 'framing'. In my understanding, most of these studies would not consider themselves as «framing studies» but more likely link to «policy design» or even policy feedback. Does considering these studies as studies about 'framing' not actually lead to a misinterpretation of the role of framing in policy preferences?**

*Thank you for this important comment. I agree that a broad conceptualization of framing requires clearer justification. In response, I now clarify more explicitly that framing is understood as the selective communication of policy-relevant information. The key point is that even studies focusing on “objective” policy attributes (such as policy design) necessarily involve choices about what information is presented, which aspects are emphasized, and how this information is formulated.*

*These are inherently framing processes, even when there is no deliberate purpose. These clarifications are now integrated into the revised manuscript in the following passages in the theory and results sections:* *“This can involve providing information about social policies’ design, recipients, outcomes and the broader context in which they are implemented. Importantly, the same information can be communicated (politically) in different ways, thereby affecting how individuals understand and evaluate social policies. For example, a benefit scheme financed through progressive taxation can be described either as imposing higher taxes on high-income earners or as levying lower taxes on low-income earners. While these descriptions refer to the same underlying policy design, citizens may respond very differently to such alternative frames .*

*In that sense, it is also worth noting that framing, as conceptualized in this article, need not be deliberate. Indeed, many of the studies included in this review do not explicitly refer to framing, but are instead situated in adjacent research traditions. This includes, most notably, research on policy design and policy feedback, which examines how features of policies shape citizens’ attitudes (Busemeyer et al., 2021), as well as work on policy recipients and welfare deservingness, which focuses on how characteristics of beneficiaries influence public support (van Oorschot, 2000). However, they all involve selecting, highlighting, and communicating particular aspects of social policies (such as their design features or recipient characteristics) in ways that may shape how citizens evaluate them, and can therefore be understood as operating through mechanisms closely related to framing”.* *“Importantly, I argue that even studies that focus on factual attributes of social policies inevitably involve choices about what information is presented to respondents, which aspects are emphasized, and how this information is formulated.*

*In that sense, such studies cannot be entirely separated from framing processes. In line with standard definitions, including the one presented above, framing operates through the selection and salience of particular aspects of reality. Applied to the studies reviewed here, this implies that presenting information about policy features–whether related to design, outcomes, recipients, or context–already entails highlighting some aspects while leaving others in the background, as well as choosing specific ways of communicating this information. Even when such studies are primarily concerned with substantive policy attributes rather than explicitly theorizing framing, they nevertheless involve the selective communication of policy-relevant information and can therefore be understood, at least implicitly, as engaging in framing.”*

**Secondly, not only is 'framing' understood very broadly, but 'social policy preferences' also means very different things in different studies. As a result, the review merges studies with very different dependent variables. This is not a bad thing as such. However, to make this heterogeneity more transparent, I suggest that the tables presenting the studies should contain information about the dependent variable and the experimental approach. This would enable readers to see more clearly what the different studies are about and draw their own conclusions. Furthermore, I believe this variety should be given more consideration when interpreting the results.**

*Thank you for this helpful comment. I agree that the studies included in the review differ substantially in their dependent variables. In response, I have made this variation a bit more explicit in the methods section by distinguishing between different types of outcome measures. I also state more clearly that I had examined this heterogeneity in my analyses (using the detailed breakdown of studies available via this* *link* *). However, this did not reveal any clear or consistent patterns linking specific types of dependent variables to particular framing effects or contingency factors.*

*For this reason, I did not treat variation in dependent variables as a central organizing dimension of the review.* *In the methods section, I now state that:* *“In terms of dependent variables, most studies focus on measurements of stated policy support, which includes support for existing social policy programs (e.g., the Earned Income Tax Credit in the US; Callaghan & Olson, 2017), reforms thereof (e.g., increasing the age required to qualify for pensions; Ingham & Levin, 2018), or entirely new policies (e.g., universal basic income; Laenen, 2023). Other studies examine preferences for increasing, decreasing, or stabilizing spending on social policy (e.g., Lergetporer et al., 2018) or preferred benefit levels (e.g., Linden & Reibling, 2022). Additionally, some studies explore support for the imposition of social obligations, such as work-related requirements (e.g., Gielens et al., 2019), benefit sanctions (e.g., Kootstra & Roosma, 2018), and administrative burdens (e.g., Haeder & Sylvester, 2024).*

*Beyond direct policy support, some studies investigate dependent variables closely related to it, such as the level of satisfaction or trust in social policy (e.g., Bendz & Oskarsson, 2024), its perceived sustainability (e.g., Goerres et al., 2020), or the perceived deservingness of policy recipients (e.g., Crabtree & Wehde, 2023). While most of the studies focus on what I call “policy-level” dependent variables, which ask about citizens’ generalized support for a social policy (e.g., Hjorth, 2016), some also have “individual-level” dependent variables, which ask about citizens’ support for granting social rights (or imposing social obligations) on individual social policy recipients (e.g., Chueri et al., 2024). In this article, however, I focus less on variation across dependent variables and more on the effects of independent variables capturing different frames, as the analysis revealed no clear patterns upon initial inspection. This nevertheless remains an important avenue for future research, as noted in the discussion section.”*

**Thirdly, and relatedly, I wonder if the author could explore whether the 'conditionalities' are systematically linked to different types of framing or dependent variables. The main conclusion that it is all about conditionalities seems realistic. However, to make this claim more pertinent and relevant, it would be important to discuss whether the many conditional and 'inconclusive' findings are really the result of conditionalities or, to a certain extent, simply the result of examining articles that ultimately study very different things. I can see two ways forward: Either the author should demonstrate more clearly that the identified conditionalities are relevant beyond specific conceptualisations of 'framing' and different dependent variables, or the author should attempt to systematise these conditionalities in relation to types of framing and/or dependent variables.**

*Thank you for this important observation. I agree that it is crucial to distinguish between genuine contingency effects and the possibility that mixed findings arise from heterogeneity in research designs and outcome variables. The evidence reviewed in this paper suggests that contingency effects are not merely an artifact of such heterogeneity. As the detailed overview table demonstrates, similar patterns recur across studies that differ substantially in their conceptualization of framing and in their dependent variables. This indicates that the identified conditionalities are robust across a wide range of empirical settings.* *At the same time, I do not think these issues can be adequately assessed within the scope of this review. Assessing how these conditionalities vary across different types of framing or outcome measures requires fit-for-purpose research designs that explicitly compare these dimensions within a single (experimental) design.*

*This point is now emphasized more clearly in the revised discussion section, where I outline how such designs could be implemented:* *“This is particularly important because answering key questions in the literature requires studies that are deliberately set up to assess the relative and combined effects of these contingency factors, and how these may vary across different types of framing–such as traditional framing, priming, persuasion, and information provision–as well as across different types of social policy preferences. Implementing such experiments would represent a significant methodological innovation, enabling researchers to capture the complex, conditional nature of framing effects on (social) policy preferences. For example, a factorial vignette experiment could simultaneously vary the type of policy preferences, the type of framing, and key contingency factors such as frame direction, format, source, and context.”*

**Minor points:**

- **What is really helpful is that specific studies are mentioned in the text to underline one or the other argument. However, the selection of these illustrative studies is somewhat unbalanced. While some studies are referenced repeatedly, others that would also fit are never mentioned.**

*Thank you for pointing out that the discussion of studies is helpful. I deliberately chose to draw more frequently on a subset of studies, as I found that repeatedly introducing new studies and experimental designs reduced the readability of the manuscript. Focusing on recurring examples allowed me to present the arguments more clearly and make the text easier to follow for readers.*

- **At several instances in the paper, formulations such as 'more recently' are used to refer to quite references that are actually quite old (or not recent, in any case).**

*Thank you for this observation. I agree that these formulations were imprecise. References described as “more recent” have now been removed or rephrased accordingly.*

- **It is not clear what time period the scoping review covers. Why are the starting and end points not defined? Some of the studies are very old, which could mean that 'outdated' findings are influencing the review's results.**

*I agree that the temporal scope of the review should be clearly specified. This information was included in an earlier version of the manuscript and has now been reintroduced in the methodology section. Regarding the inclusion of older studies, I acknowledge that a handful of experiments date back to the 1990s. However, it is not straightforward to determine from which point findings would be considered “outdated,” and several of these earlier studies remain foundational to the literature. For this reason, I adopt an inclusive approach while making the temporal distribution of studies explicit in the manuscript. This information is also included in the detailed breakdown per study, attached to this response.* *In the methods section, I have now added the following passage:* *“With regard to the time period, most of the experiments included in this review were conducted after 2010. There are fewer experiments from before 2010, and only a handful from the 1990s. The earliest experiments discussed here were all conducted in the US, either as part of the extensively analyzed 1986 American National Election Study (ANES) Pilot (see Iyengar, 1990; Groskind, 1991, 1994; Ponza et al., 1988; Will, 1993), or within the framework of the classical question wording experiments contrasting public support for "welfare" and "assistance to the poor" (Smith, 1987; Huber & Paris, 2023).”*

- **The tables are important, but they are also very long and difficult to read. Perhaps consider including summary tables in the main text and moving the long tables to the appendix.**

*Thank you for this helpful suggestion. The detailed tables have now been moved to the appendix and replaced in the main text with more accessible summary figures.*

- **The criticism that many studies do not examine subgroup differences seems to be phrased rather harshly. There may be reasons why subgroup differences are not relevant or have not been studied. Alternatively, they may have been studied, but no heterogeneity was found.**

*I agree that the original phrasing was too strong. I have revised this passage to adopt a more balanced tone, explicitly acknowledging that there may be valid reasons why subgroup differences are not examined or reported, while still highlighting the implications this may have for interpreting framing effects (see revised passage below).* *“This leads to a final, critical point about the influence of receiver characteristics: the frequently limited attention given to heterogeneity in treatment effects. Much of the literature on the framing of social policy preferences reports only average treatment effects across the full sample (i.e., 42 studies in this review), which may obscure important subgroup variation.*

*While there may be valid reasons (such as theoretical expectations, data limitations, or the absence of observed heterogeneity), this omission nonetheless carries two main risks. First, it may create the impression that a framing effect is universal when it actually applies only to specific subgroups. Racial framing effects, for instance, are often limited to individuals with particularly negative priors toward ethnic minorities and migrants, such as radical right-wing voters (see above). Second, average treatment effects can lead to potentially misleading conclusions when opposing subgroup responses cancel each other out. For instance, some studies (e.g.,* *Avdagic & Savage, 2024* *) show that left-wing and right-wing voters sometimes move in opposite directions in response to the same information, which could lead to the erroneous conclusion that the frame is ineffective if subgroup dynamics are not taken into consideration.”*

# Reviewer report: Reviewer response for version 1

**DOI:** 10.21956/openreseurope.23364.r65645

**Published:** 2025-12-30

**Authors:** Marius R Busemeyer

**Affiliations:** 1 University of Konstanz, Konstanz, Germany

This paper provides a comprehensive and differentiated review of the vast literature on the effects of framing on social policy attitudes. This literature is to some extent fragmented due to different concepts used and the fact that papers usually employ their own experimental designs in the empirical analysis. Thus, this paper could be a very useful reference point as the field develops further.

 I have a few remarks and suggestions for improvement. 

 First, although framing is different from policy feedback, there are obvious connection points, e.g. when the paper discusses the role of context in shaping framing effects. In fact, exploring these connections deeper could be an interesting avenue for future research. The paper could and should acknowledge this link more explicitly and give a short nod to the equally extensive policy feedback literature.

 Second, I appreciate and like to careful sorting and definition of framing effects into the categories of priming, framing and information provision (p. 4). Unfortunately, these distinctions get lost in the further development of the argument, but they should be strengthened. For instance, I would be very interested in a discussion about whether politically motivated framing (or priming) effects are stronger than simple information provision effects - again, an interesting point for future research.

 Third, the paper spends most time on discussing the "conditions" under which framing effects matter or not. A large part in this section is the discussion of "content" of framing. I find this part to some extent unsatisfactory. The main problem/challenge here is that not all aspects related to policy design can and should be understood as framing effects. I see that content-related issues should also be part of the discussion about framing, but not all questions related to policy design asked in survey experiments are automatically framing effects. The paper should discuss this issue more explicitly and develop an argument on how to distinguish between framing-related design effects and simple design effects. Why this is important? If one adopts a broad understanding of framing effects (i.e. any issues related to policy design), then of course the literature that would need to be reviewed is even more extensive.

 Fourth, even though I share the paper's main conclusion supporting a contingent view on framing effects, the conclusion remains somewhat underwhelming (also because the extreme positions have certain strawman-like qualities). It would be helpful to be a bit more precise in  spelling out in greater detail which conditions matter and why as well as which aspects of framing (out of the four possible ones mentioned in the paper) are more important.

 With regard to presentation, the paper is quite densely written and at times hard to follow. I would suggest to have even more sub-sections in order to guide the reader. Also the very long tables are hard to process and read. There should be ways of presenting this in a more accessible manner, e.g. using bar charts or figures with the percentages. As common in meta-reviews, it could also be possible to run simple regressions (DV=framing effect (yes/no), IVs=the various aspects related to framing) in order to tease out the underlying patterns.

Are all factual statements correct and adequately supported by citations?

Yes

Is the review written in accessible language?

Partly

Are the conclusions drawn appropriate in the context of the current research literature?

Yes

Is the topic of the review discussed comprehensively in the context of the current literature?

Yes

Reviewer Expertise:

Comparative welfare state research, comparative political economy, public attitudes towards the welfare state, political and socio-economic inequality

I confirm that I have read this submission and believe that I have an appropriate level of expertise to confirm that it is of an acceptable scientific standard, however I have significant reservations, as outlined above.

# Response

**Published:** 2026-07-08

**Authors:** Tijs Laenen

**Affiliations:** Sociology, Antwerp University Faculty of Political and Social Sciences, Antwerp, Flanders, Belgium

**This paper provides a comprehensive and differentiated review of the vast literature on the effects of framing on social policy attitudes. This literature is to some extent fragmented due to different concepts used and the fact that papers usually employ their own experimental designs in the empirical analysis. Thus, this paper could be a very useful reference point as the field develops further.**

**I have a few remarks and suggestions for improvement.**

**First, although framing is different from policy feedback, there are obvious connection points, e.g. when the paper discusses the role of context in shaping framing effects. In fact, exploring these connections deeper could be an interesting avenue for future research. The paper could and should acknowledge this link more explicitly and give a short nod to the equally extensive policy feedback literature.**

*Thank you for this insightful suggestion. I fully agree that there are important conceptual connections between the framing literature and policy feedback research. In response, I have now made this connection more explicit in both the theory and discussion sections. In the theory section, I clarify that many studies in adjacent traditions (most notably policy feedback theory, but also deservingness theory) can be understood as engaging with mechanisms closely related to framing, insofar as they involve the selective communication of policy features (i.e., design and recipients, respectively).*

*In the discussion section, I further develop this point by explicitly linking the proposed framework to these research traditions.* *More specifically, the following is added to the theoretical section:* *“This can involve providing information about social policies’ design, recipients, outcomes and the broader context in which they are implemented. Importantly, the same information can be communicated (politically) in different ways, thereby affecting how individuals understand and evaluate social policies. For example, a benefit scheme financed through progressive taxation can be described either as imposing higher taxes on high-income earners or as levying lower taxes on low-income earners. While these descriptions refer to the same underlying policy design, citizens may respond very differently to such alternative frames. In that sense, it is also worth noting that framing, as conceptualized in this article, need not be deliberate. Indeed, many of the studies included in this review do not explicitly refer to framing, but are instead situated in adjacent research traditions. This includes, most notably, research on policy design and policy feedback, which examines how features of policies shape citizens’ attitudes (Busemeyer et al., 2021), as well as work on policy recipients and welfare deservingness, which focuses on how characteristics of beneficiaries influence public support (van Oorschot, 2000).*

*However, they all involve selecting, highlighting, and communicating particular aspects of social policies (such as their design features or recipient characteristics) in ways that may shape how citizens evaluate them, and can therefore be understood as operating through mechanisms closely related to framing”.* *And the following is added to the discussion section:* *“Furthermore, the framework speaks to related strands of research beyond the framing literature. One prominent example is policy feedback theory (Busemeyer et al., 2021), which examines how policy design influences citizens’ attitudes, and how these effects are conditioned by who communicates policy features, who receives them, how they are communicated, and the context in which this occurs. Likewise, it informs welfare deservingness theory (van Oorschot, 2000) by highlighting that citizens’ evaluations of policy recipients are contingent on how their characteristics are communicated and interpreted. Future research within these traditions could more explicitly investigate how citizens’ responses to information about social policies’ design and recipients are shaped by the interplay between the contingency factors highlighted here”.*

**Second, I appreciate and like to careful sorting and definition of framing effects into the categories of priming, framing and information provision (p. 4). Unfortunately, these distinctions get lost in the further development of the argument, but they should be strengthened. For instance, I would be very interested in a discussion about whether politically motivated framing (or priming) effects are stronger than simple information provision effects - again, an interesting point for future research.**

*Thank you for this important remark. I agree that the distinction between different types of communicative interventions (i.e., framing, priming, persuasion and information provision) is theoretically meaningful and should be taken seriously. At the same time, the review does not allow for firm conclusions regarding their relative strength or effectiveness. As the extended study-by-study overview table demonstrates (available via this* *link* *), existing studies differ substantially in how these concepts are operationalized, often combining multiple elements within a single experimental design and rarely allowing for direct comparison across intervention types. For this reason, I argue that answering this question requires studies that are explicitly designed to compare these mechanisms within a unified framework. I have therefore incorporated this point into the discussion section as a key direction for future research:* *“To the best of my knowledge, no existing study has employed such a design, despite the clear signals that framing effects are shaped by interactions between frames, senders, receivers, and context. This is particularly important because answering key questions in the literature requires studies that are deliberately set up to assess the relative and combined effects of these contingency factors, and how these may vary across different types of framing–such as traditional framing, priming, persuasion, and information provision–as well as across different types of social policy preferences.*

*Implementing such experiments would represent a significant methodological innovation, enabling researchers to capture the complex, conditional nature of framing effects on (social) policy preferences. For example, a factorial vignette experiment could simultaneously vary the type of policy preferences, the type of framing, and key contingency factors such as frame direction, format, source, and context.”*

**Third, the paper spends most time on discussing the "conditions" under which framing effects matter or not. A large part in this section is the discussion of "content" of framing. I find this part to some extent unsatisfactory. The main problem/challenge here is that not all aspects related to policy design can and should be understood as framing effects. I see that content-related issues should also be part of the discussion about framing, but not all questions related to policy design asked in survey experiments are automatically framing effects. The paper should discuss this issue more explicitly and develop an argument on how to distinguish between framing-related design effects and simple design effects. Why this is important? If one adopts a broad understanding of framing effects (i.e. any issues related to policy design), then of course the literature that would need to be reviewed is even more extensive.**

*I agree that the relationship between framing and policy design requires clearer conceptual justification. In the revised manuscript, I have strengthened the argument that studies focusing on policy design cannot, in my view, be entirely separated from framing processes. Specifically, I now clarify that even when experiments aim to capture the effects of ‘objective’ policy attributes, they necessarily involve choices about which aspects are presented, how they are formulated, and which dimensions are made salient. In this sense, framing is understood not as equivalent to policy design, but as operating through the selective communication of policy-relevant information. This distinction is now made more explicit in both the theoretical discussion (see above, in my response to the reviewer’s first point) and the results section:* *“Importantly, I argue that even studies that focus on factual attributes of social policies inevitably involve choices about what information is presented to respondents, which aspects are emphasized, and how this information is formulated. In that sense, such studies cannot be entirely separated from framing processes. In line with standard definitions, including the one presented above, framing operates through the selection and salience of particular aspects of reality. Applied to the studies reviewed here, this implies that presenting information about policy features–whether related to design, outcomes, recipients, or context–already entails highlighting some aspects while leaving others in the background, as well as choosing specific ways of communicating this information. Even when such studies are primarily concerned with substantive policy attributes rather than explicitly theorizing framing, they nevertheless involve the selective communication of policy-relevant information and can therefore be understood, at least implicitly, as engaging in framing.”*

**Fourth, even though I share the paper's main conclusion supporting a contingent view on framing effects, the conclusion remains somewhat underwhelming (also because the extreme positions have certain strawman-like qualities). It would be helpful to be a bit more precise in spelling out in greater detail which conditions matter and why as well as which aspects of framing (out of the four possible ones mentioned in the paper) are more important.**

*I agree that it would be valuable to provide more precise conclusions regarding which conditions matter most and which aspects of framing are most influential. However, the evidence reviewed in this article does not allow for such stark conclusions. As the detailed overview of studies demonstrates, most experiments isolate only a limited number of factors and do not systematically vary multiple dimensions of framing within the same design. As a result, it is not possible to assess the relative importance of different contingency factors or to compare their effects directly. For this reason, I argue that addressing these questions requires a new generation of studies that are deliberately designed to examine the relative and combined effects of frame, sender, receiver, and context characteristics within a single framework. This point is now emphasized more clearly in the discussion section as a central implication of the review.*

**With regard to presentation, the paper is quite densely written and at times hard to follow. I would suggest to have even more sub-sections in order to guide the reader. Also the very long tables are hard to process and read. There should be ways of presenting this in a more accessible manner, e.g. using bar charts or figures with the percentages. As common in meta-reviews, it could also be possible to run simple regressions (DV=framing effect (yes/no), IVs=the various aspects related to framing) in order to tease out the underlying patterns.**

*Thank you for these suggestions. I agree that improving the accessibility and readability of the manuscript is important, especially given the breadth of the literature reviewed. In response, I have made several changes to improve the structure and presentation of the paper. First, I have introduced additional sub-sections throughout the manuscript to guide the reader more clearly through the argument. Second, I have replaced parts of the presentation of the findings with more accessible visual summaries, including figures, while moving the overview tables to the appendix.* *Regarding the suggestion to conduct regression analyses on the reviewed studies, I agree that this is a common strategy in meta-analyses. However, I do not believe that such an approach is appropriate in the context of this review.*

*The studies included in the analysis differ substantially in terms of their conceptualization of framing, operationalization of independent variables, outcome measures, and research designs. As a result, coding a dichotomous dependent variable such as the presence or absence of a framing effect would require strong simplifying assumptions and would risk obscuring the substantive differences between studies. More importantly, many of the key questions raised in this review (such as the relative importance of different contingency factors or their interactions) cannot be meaningfully assessed through ex-post comparisons across heterogeneous studies. Instead, addressing these questions requires experimental designs that are explicitly and deliberately set up to vary multiple factors simultaneously. I therefore emphasize in the discussion section that future research should prioritize such designs.*
